# Transfer hydrogenation of ketone; an *in situ* approach toward an eco-friendly reduction†

**DOI:** 10.1039/d2ra02701a

**Published:** 2022-07-07

**Authors:** Oluwatayo Racheal Onisuru, Dele Peter Fapojuwo, Charles O. Oseghale, Oyekunle Azeez Alimi, Reinout Meijboom

**Affiliations:** Department of Chemical Sciences, University of Johannesburg P.O. Box 524, Auckland Park Johannesburg 2006 South Africa rmeijboom@uj.ac.za +27 (0)11 559 2819 +27 (0)72 894 0293

## Abstract

The use of water as a solvent in chemical reactions has recently been brought to public attention, especially in the exploration of eco-friendly procedures. It is readily available, abundantly accessible, non-toxic, non-flammable, and at a low cost. As opposed to the previous limitation of reactant solubilities associated with aqueous media, a hydrogel such as a hydroxypropyl methylcellulose (HPMC) solution can significantly improve the reactant solubility. This investigation employed water and HPMC as the reaction solvent, and the reaction medium viscosity was impressively enhanced. Silica-supported Pd particles (Pd@SiO_2_) were synthesized and effectively catalyzed the reduction of acetophenone in the presence of sodium borohydride (NaBH_4_) as the hydrogen source. The conversion of acetophenone to 1-phenyl ethanol remained at a very high value of >99.34% with 100% selectivity towards 1-phenyl ethanol.

## Abstract 2

The use of water as a solvent in chemical reactions has recently been brought to public attention, especially in the exploration of eco-friendly procedures.

## Graphical abstract

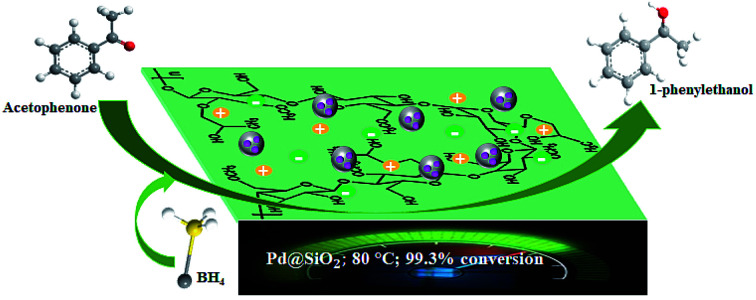

## 1. Introduction

Water's versatility as a chemical system solvent is demonstrated in its relatively high polarity and high specific heat. This versatility is in addition to water's high dielectric constant, surface tension, and significant energy density.^1,2^ This is so as water molecules' charge is unevenly distributed, making different charged and polar molecules dissolvable in water.^2^ One of the fundamentals of modern chemistry which has a key role in global sustainability is the generation of the final product without additional harmful substances.^3^ This is dependent not only on the choice of catalyst but also on the solvent. Water has been reported as an interesting greener solvent that enhances organic synthesis.^4^ Its general availability, affordability, incombustibility, and inexplosive nature made it a perfect replacement for organic solvents in the development of practical reactions.^1,5–7^ The hydrogen bonding permits the molecular groups to generate high heat of vaporization and polarization of water. These characteristic features bring about electron transfer as an essential process in water-mediated reactions. Also, these features are in addition to the cohesive, adhesive, and viscous nature of water that enables facile extractive workup and purification of the product.^5,8^ These physical and chemical properties (ability to form hydrogen bonds and the amphoteric nature) of water help to theoretically enhance chemical reactions in terms of the reactivity and selectivity in water.^1^ In recent years, water-tolerant catalysts and water-soluble ligands with excellent efficiency and stereoselectivity have been developed for organic reactions in water.^9^

Hydroxypropyl methylcellulose (HPMC) is a group of cellulose ester that has substituted one or more of the hydroxyl groups occupying the cellulose ring with hydroxypropyl and methyl groups.^10,11^ It is a water-soluble (hydrophilic), environmentally friendly, and biodegradable polymer. It has industrial applications ranging from pharmaceutical,^12^ agricultural to textiles (dye, paints), cosmetics,^10^ and to coating or adhesive.^13^ Because of its edibility, its usefulness in food industries includes stabilization and emulsification. This is owing to its odorless, tasteless, non-toxic, and biocompatibility features.^14,15^ Therefore, the HPMC has an unparallel application in the aspect of environmentally benign protocols for mild reaction conditions. It is available, cheap, renewable, and possesses relevant film-forming features that generate mechanical activity.^13,15^

Although hydroxypropyl methylcellulose is a temperature-sensitive biopolymer that forms a gel upon heating between 75 and 90 °C, it also has a dissolution tendency in cold organic solvents of polar nature. Hence, these properties make it relevant for use in both aqueous and non-aqueous solvents. Both hydroxypropyl (polar) and methyl (non-polar) groups enable interaction within the intermolecular, intramolecular, and hydrophobic environments.^14^ Thus, the combined activities of water and HPMC thermal gelation property^16^ are capable of accelerating aqueous reactions, especially for insoluble organic chemical reactions by improving the reaction medium solubility.

Modern organic synthesis requires that attention be placed on selectivity related to the control of complete stereochemistry, reactivity, and productivity.^17^ Sustainability and eco-friendliness are part of keywords or concepts which have put chemical synthesis and process chemistry into perspective.^3^ This is because of chemists' quest for environmental protection, and safer design of molecules, materials, products, and processes which have increased.^18^ The unrenewable source, cost ineffectiveness in production and disposal, including the human and ecological hazard of most organic solvents have put them on the spot, hence making the need to find a replacement for organic solvents more pressing.^18^ One fundamental development in synthetic chemistry is the selectivity of organic transformation through hydrogenation.^5,19^ This process reduces the amount of materials consumed, leading to redundant trimming and reactions' step re-functionalization.^5,20^ Transfer hydrogenation is one of the most cardinal organic transformations, which entails the substitution of hydrogen with a molecule of non-H_2_ possessing derivative.^21^ There has not been a report on the transfer hydrogenation of acetophenone investigated using HPMC solution to the best of our knowledge.

**Scheme 1:**
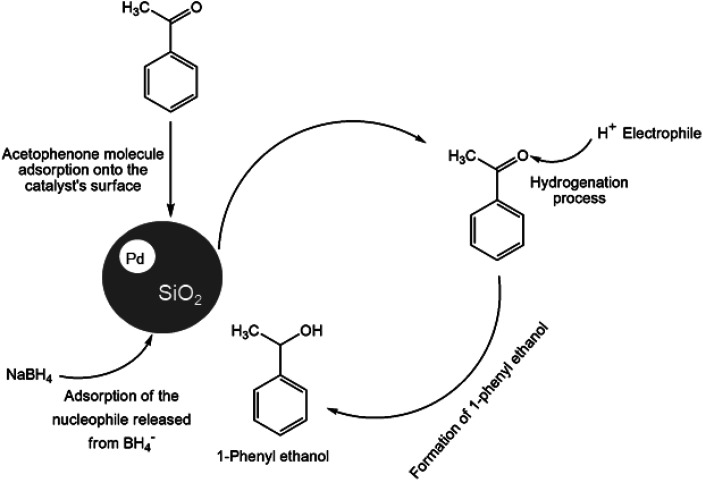
Schematic representation of 1-phenyl ethanol formation.

In recent years, the industrial application of ketone hydrogenation has received wide attention as a diverse range of valuable alcohols, and chiral compounds are produced.^22^ The great significance of acetophenone hydrogenation is in the synthetic relevance of the chemical intermediates it generates, including 1-phenyl ethanol, ethylbenzene, and cyclohexyl ethanol.^23,24^ Transfer hydrogenation of acetophenone being a model reaction is owing to acetophenone's hydrogenative groups, namely the phenyl group and the carbonyl group, which are reduced to their corresponding alcohol.^25^ The (C=O) double bond of the carbonyl group is hydrogenated to produce 1-phenyl ethanol, an essential chemical intermediate used in the fragrance, flavor, and pharmaceutical industry.^26,27^ However, the phenyl group hydrogenation yields a cyclohexyl methyl ketone.^28^ In this work, the combined advantage of HPMC hydrogel and water was explored for the *in situ* hydrogenation of ketones to generate alcohol as shown in the reaction pathway in Scheme 1 without side or unwanted products. This may be an additional convectional procedure for industrial application.

There have been reports on Pd-based materials' practical application as catalysts for ketone hydrogenation.^29–31^ Pd nanoparticles' superior catalytic performance, in organic transformation and hydrogen adsorption capability even at room temperature have been asserted in other chemical other reactions.^32,33^ Pd/SiO_2_–Al_2_O_3_ catalysts were successfully used to catalyze acetophenone, but the primary product generated was ethylbenzene with this material. However, silica-supported Pd particles (Pd@SiO_2_) can be used as a potentially effective catalyst^29^ to reduce acetophenones into an eco-friendly species.

## 2. Experimental section

### 2.1 Chemicals and materials

The chemicals used were obtained from commercial suppliers of different companies and were of analytical grades. Hydroxypropyl methylcellulose (HPMC) average Mn ∼10 000, ethanol (99.9%) was obtained from Monitoring Control Laboratories Ltd, palladium(ii) acetate (98%) (C_4_H_6_O_4_Pd), tetraethyl orthosilicate (TEOS) (99%), and acetophenone (99%) (C_8_H_8_O) were bought from Sigma-Aldrich. *n*-Decane, (CH_3_(CH_2_)_8_CH_3_) (99%) was purchased from Alfa Aesar, magnesium sulphate dried (MgSO_4_) (52–70%) was obtained from Rochelle chemicals, sodium borohydride (NaBH_4_) (≥98.0%) was purchased from Sisco Research Laboratories (SRL) Pvt. While ethyl acetate, sodium bicarbonate NaHCO_3_ (99.50%), and NaOH (99.90%) were obtained from Promack chemicals. Milli-Q water of 18.2 MΩ cm was used to prepare all the solutions.

### 2.2 Synthesis of silica support for the Pd particle and impregnation of Pd unto the silica support

The silica support was synthesized following a literature procedure.^34^ Typically, aqueous (11.20 mL) NH_4_OH (27 wt%) was added to a mixture of ethanol (200 mL) and deionized water (50 mL) at room temperature and was allowed to stir for 30 min. After that, 8.93 mL of TEOS was added drop by drop to the mixture. It was further stirred for 12 h, and silica precipitation was observed. The formed silica precipitate was obtained through centrifugation, washed with deionized water, and centrifuged into pellets. The pellet was left to dry overnight in the oven at 70 °C to obtain dry silica, and it was later calcined at 500 °C.

The silica support was dispersed in 8 mL of deionized water. A solution of Pd acetate containing 0.0212 g was added to the slurry, and it was left to stir for 4 h. Sodium borohydride (8 mL) was used to reduce the metal ion, and it was allowed to stir for another 4 h, and it was collected after centrifuging the slurry. The solid residue was dried overnight in the oven at 70 °C, and it was calcined for 4 h at 150 °C. After that, the dried product (Pd@SiO_2_) obtained was ground, characterized, and catalytically tested. Inductively coupled plasma optical emission spectroscopy (ICP-OES) was used to determine the amount of Pd loaded on the silica (0.52 wt%).

### 2.3 Catalyst characterization

The characterization techniques carried out include Brunauer–Emmett–Teller (BET) nitrogen sorption measurement of the samples using a Micromeritics ASAP 2460 instrument to get the surface area. At the same time, the Barrett–Joyner–Halenda (BJH) desorption isotherm was used to obtain the pore size and volume after degassing the sample at 100 °C to constant weight. Powder X-ray diffraction (p-XRD) patterns were performed at room temperature using a Rigaku SmartLab at a 2*θ* range from 5° to 80° angle with Cu Kα radiation (*λ* = 1.54056 Å). High-resolution transmission electron microscopy (HR-TEM) images were captured at 200 kV accelerating voltage using a JEOL JEM-2100F electron microscope. A certain amount of the catalyst was suspended in methanol ultrasonically, then a few drops were deposited on the carbon-coated grid and were allowed to dry before the analysis. Tescan Vega 3 LMH Scanning Electron Microscope (SEM) with a 20.0 kV of electron scattering detector voltage was used to acquire the catalyst's morphology. The sample was carbon-coated before the image capturing, using an Agar Turbo Carbon Coater. For the execution of the catalyst's reducibility analysis, hydrogen temperature-programmed reduction was performed using a Micromeritics Autochem II 2920 by probing with 10% H_2_/Ar at a flow rate of 50 mL min^−1^ within the range of 25–800 °C having 10 °C min^−1^ ramp rate. After an argon flow purged the catalyst's surface for 1 h at 200 °C, the analysis was carried out. Fourier transform infrared spectroscopy (FTIR) was conducted on Shimadzu IRAffinity-1 to identify the specific functional groups of the sample's chemical composition. Thermal analysis was done by subjecting a certain amount of the catalyst to a temperature range from 25 °C to 1000 °C with a heating ramp rate of 10 °C min^−1^ under a nitrogen flow of 100 mL min^−1^ flow rate on a thermogravimetric analyzer (TGA) PerkinElmer STA 6000.

### 2.4 Transfer hydrogenation of ketone in HPMC solution

In a typical procedure, the reduction proceeded by pipetting 21.6 mmol acetophenone into a 50 mL carousel tube containing Pd@SiO_2_ (0.22 g), and 0.5 mmol of decane. A certain amount of NaBH_4_ and a 6 mL solution of hydroxypropyl methylcellulose (HPMC) prepared by dissolving 2% HPMC in 100 mL of water at 80 °C were also added. The reaction was allowed to stir continuously at 600 rpm and 80 °C for a duration of 30 min, 1 h, and 2 h separately and repeatedly on a multi-reactor (Radley Discovery Technologies) connected to a reflux condenser. Product extraction was done using ethyl acetate to separate the organic layer at the end of each catalytic run, and the extracts were passed through a column of MgSO_4_ anhydrous. Spectrophotometric analysis of the substrate (acetophenone) and the product (1-phenyl ethanol) was monitored and obtained on a Shimadzu ultraviolet visible UV-1800 in a 3 mL quartz cuvette. The absorbance was obtained from *λ* 700 nm to *λ* 250 nm. The product was analyzed using a gas-chromatography GC-2010 plus (Shimadzu) connected with a flame ionization detector (FID) and a Restek-800-356-1688 capillary column of 30 m × 0.25 mm × 0.25 μmol film thickness. While the injection port was fixed at 250 °C, the FID temperature was set at 300 °C. Besides, the carrier gas used was nitrogen gas, and the temperature of the column remained at 300 °C. The product identification was acquired through a Shimadzu GC-MS QP2010, which uses a fused silica capillary column of 30 m × 0.25 mm × 0.25 μmol film thickness.

## 3. Result and discussion

### 3.1 Characterization

**Fig. 1:**
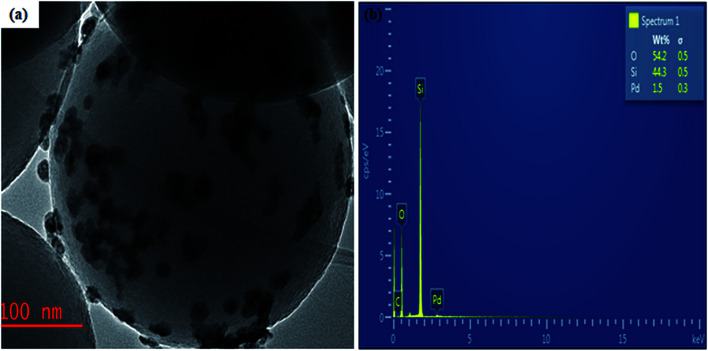
(a) TEM image obtained for Pd@SiO_2_ and (b) is the EDS showing the composition of the Pd@SiO_2_.

The TEM analysis performed to confirm the internal morphology of Pd@SiO_2_ is presented in Fig. 1a. The image revealed a well synthesized, spherical structure silica framework with a surface that enhances the impregnation of the Pd particles. The metal particles are also shown to be loaded on the silica material. Hence, this conforms with the previously reported result.^35^ The electron dispersion spectroscopy (EDS) analysis obtained from SEM/EDS is shown in Fig. 1b, while the SEM result showing the surface morphology Pd@SiO_2_ is presented in Fig. S1 (ESI†). The EDS revealed the different energy spectrums of the specific elements present in the material. Palladium element displayed minimal energy spectra compared to other elements due to the small amount (0.52 wt% as obtained from the ICP-OES analysis), of Pd impregnated on the support. Also, the carbon (C) shown in the result is due to the carbon coating that was performed before the analysis.

**Fig. 2:**
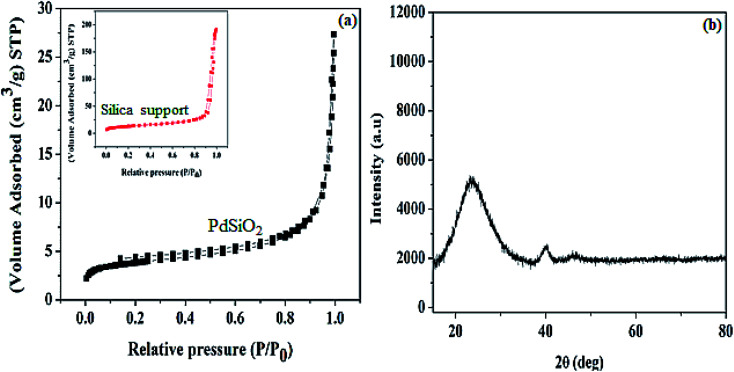
(a) N_2_ adsorption–desorption isotherm of Pd@SiO_2_, (b) powder-XRD pattern of Pd@SiO_2_.

The result N_2_ adsorption–desorption curve obtained for Pd@SiO_2_ is shown in Fig. 2a. The result indicates that an adsorption isotherm of a type I hysteresis loop is seen based on the international union of pure and applied chemistry (IUPAC) classification.^36^ This is an indication of typical non-porous material. The surface area obtained (13.14 m^2^ g^−1^) between the pressure range 0.91 and 1.00 *P*/*P*_0_ is similar to the previously reported result.^34^ In addition, the surface area obtained for the silica support (44.91 m^2^ g^−1^) was acquired between the pressure range of 0.89 and 0.99 *P*/*P*_0_. We observed that the surface area decreased after the impregnation of the Pd metal. We deduced that the surface area change is due to the surface coverage of the silica support after Pd impregnation.

The p-XRD pattern of the material is shown in Fig. 2b. The diffraction peak centered at 2*θ* = 23° can be ascribed to amorphous silica.^37^ The peaks observed at 2*θ* = 40.2° and 46.6° are exhibited by the Pd diffraction peak, and they correspond to the face-centered plane of (111) and (200), respectively.^29,38^ In this case, the Pd peak obtained indicates well-dispersed homogenized particles on the support material as revealed by the TEM image obtained.

**Fig. 3:**
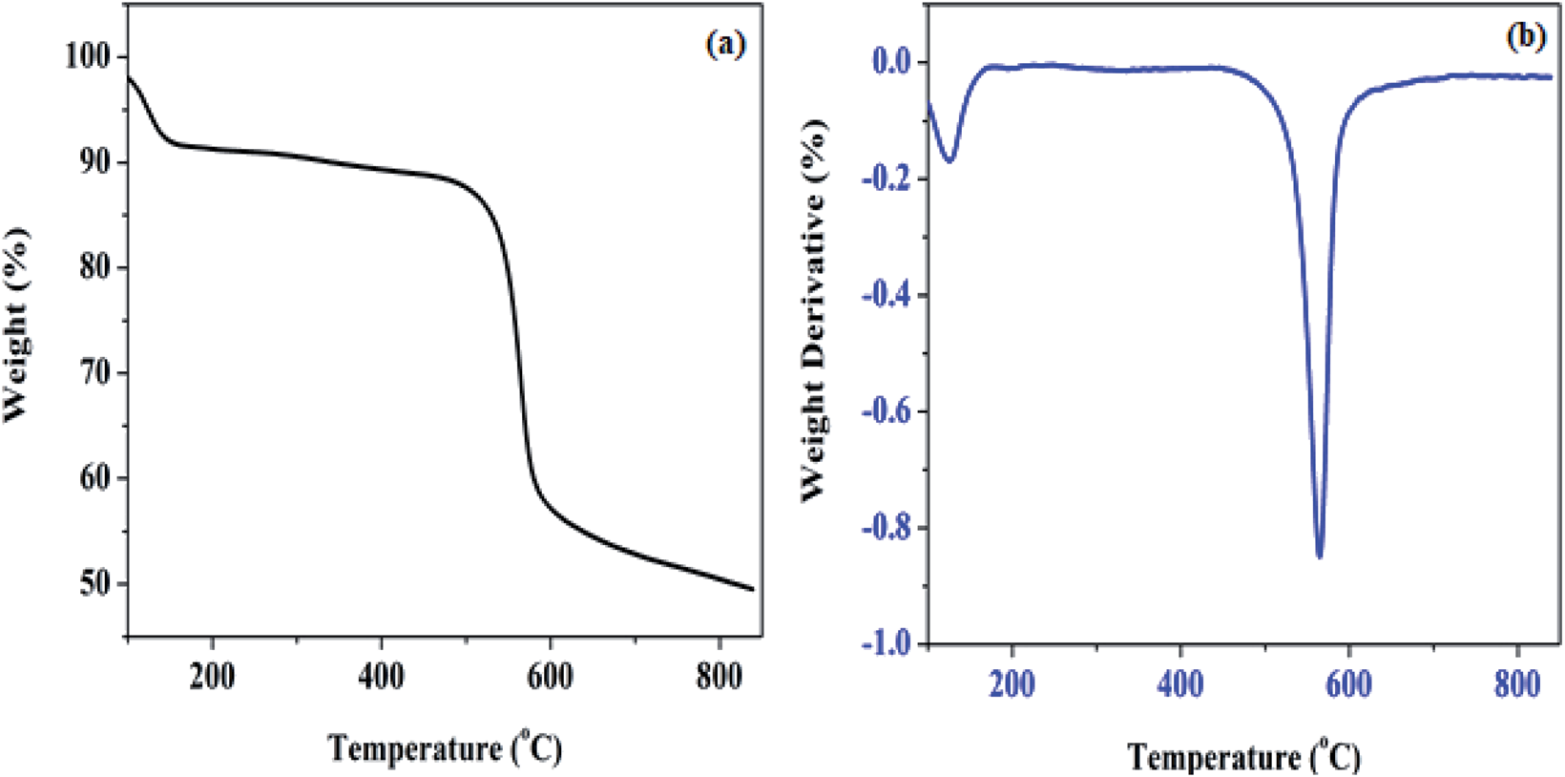
Thermogravimetric analysis of Pd@SiO_2_ (a) percentage weight loss, (b) differential weight.

The thermogravimetric analysis of Pd@SiO_2_ and its differential profile (TG/DTG) measured as the temperature changes over time are shown in Fig. 3. The thermogram profile in Fig. 3a demonstrated two different weight loss processes. The first degree of degradation between 0 and 109 °C is an indication of surface absorbed hydroxyl loss, which appearance is connected to moisture adsorption since calcination was performed in air. Also, exposure to the atmospheric air before the analysis was conducted is another factor responsible for the moisture. Moreover, the second weight loss recorded is attributed to condensed silanol decomposition, which aggregates into oxides on the silica surface.^37^ Amorphous silica hydrolyzed by the disintegration of siloxane bond (≡Si–O–Si≡). Also, the differential curve in Fig. 3b shows the step where the weight loss was maximum, which was prominently displayed at the second degradation stage.

**Fig. 4:**
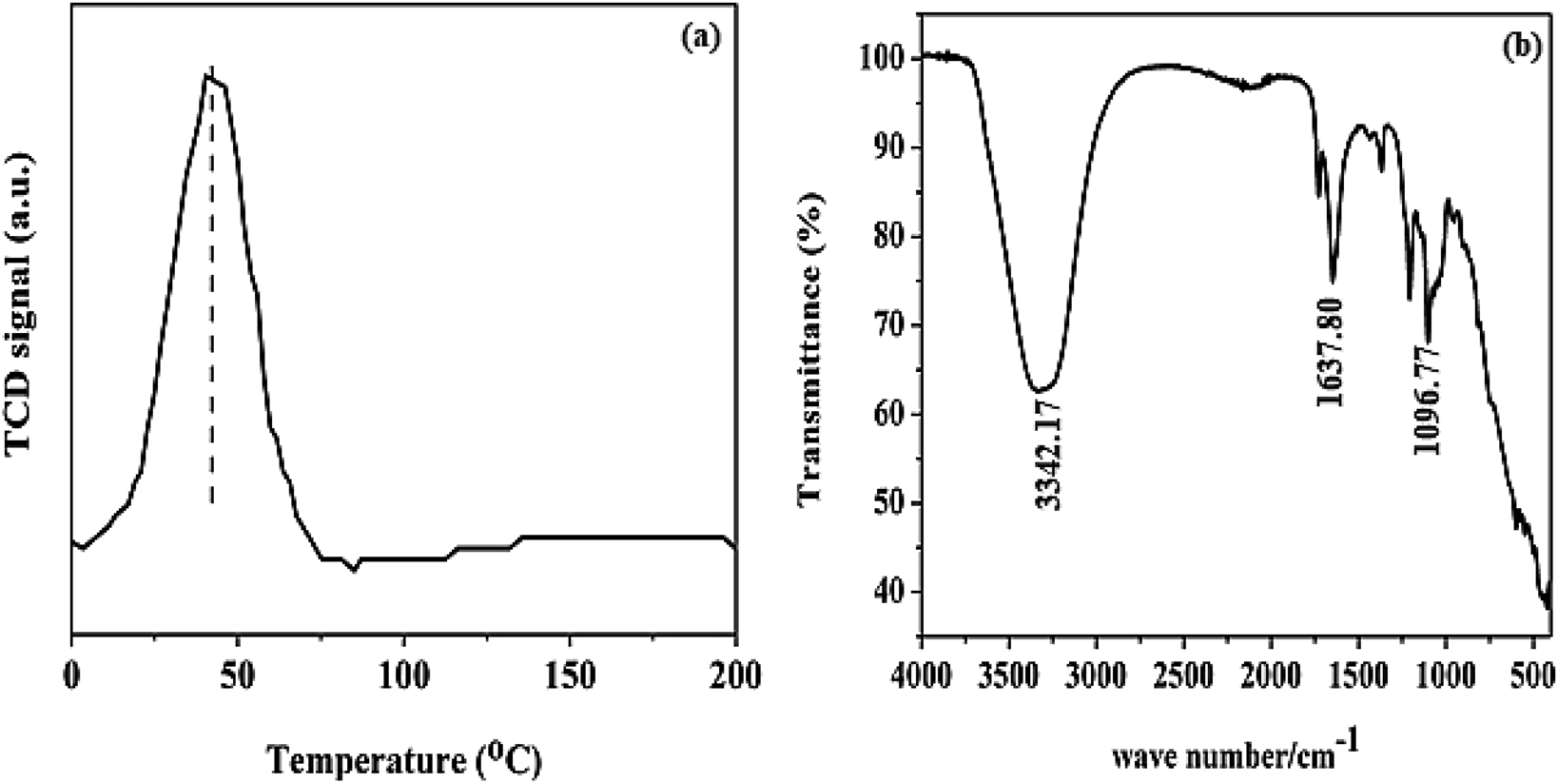
(a) H_2_-temperature program reduction analysis (b) FTIR profile of Pd@SiO_2_.

The H_2_-TPR analysis result shown in Fig. 4a was carried out to verify the reduction profile of Pd@SiO_2_. The profile revealed that Pd@SiO_2_ exhibits only one reduction peak, mainly around 45 °C. This peak can be ascribed to the reduction peak of oxidized palladium species (PdO) present in Pd@SiO_2_.^38,39^

The FTIR spectrum of Pd@SiO_2_ in Fig. 4b displayed both broad and sharp bands. The bands at 3342 and 1637 cm^−1^ are associated with H–O–H asymmetric vibration due to moisture in the material.^40^ The sharp peak that appeared at 1096.77 cm^−1^ is ascribed to the asymmetric vibration of Si–O–Si.^41^

### 3.2 Transfer hydrogenation of ketone in HPMC solution

The mesoporous-based metal oxide catalysts were screened for the transfer hydrogenation of acetophenone to select the best active catalyst for the reaction. The catalysts used are manganese metal oxide (MnMMO), cobalt metal oxide (CoMMO), nickel metal oxide (NiMMO), zinc metal oxide (ZnMMO), zirconium metal oxide (ZrMMO), and iron metal oxide (FeMMO), but no reaction occurred (result not shown). However, the minor effect of BH_4_^−^ on the substrate was observed, indicating that the reaction is catalytically dependent.

The reduction process of acetophenone is a complex reaction due to the two competing groups (carbonyl and phenyl groups) within the ketone molecule.^42,43^ The catalytic reduction of acetophenone using Pd@SiO_2_ formed 1-phenyl ethanol, a useful intermediate in the food (flavor) and cosmetics (fragrance) industry. The percentage yield conversion of acetophenone and other substrates to the product was analyzed using (Equation ESI) eqn (S1),† while the selectivity was computed with eqn (S2).† The results obtained demonstrated that support material is a crucial tool in modifying the hydrophobicity of the surface character of the support.^28^ Previously, it was reported that the influence of conversion in the selectivity and catalytic activity depends not only on the solvent effect, metal particle, and electronic properties but also on the metal–support material and their interaction.^44,45^ The presence of silanol with a peculiar feature of affinity for water, as seen in the TGA and FTIR analysis, enhanced a hydrophilic effect upon the catalyst's surface through adsorption of the hydroxyl group and other polar molecules,^28^ which enhanced the selectivity and the product yield. In addition, the more substantial contributing effect of HPMC at higher temperatures through phase restructuring, miscibility, and interconnected viscosity brought an incredible transformation.

**Fig. 5:**
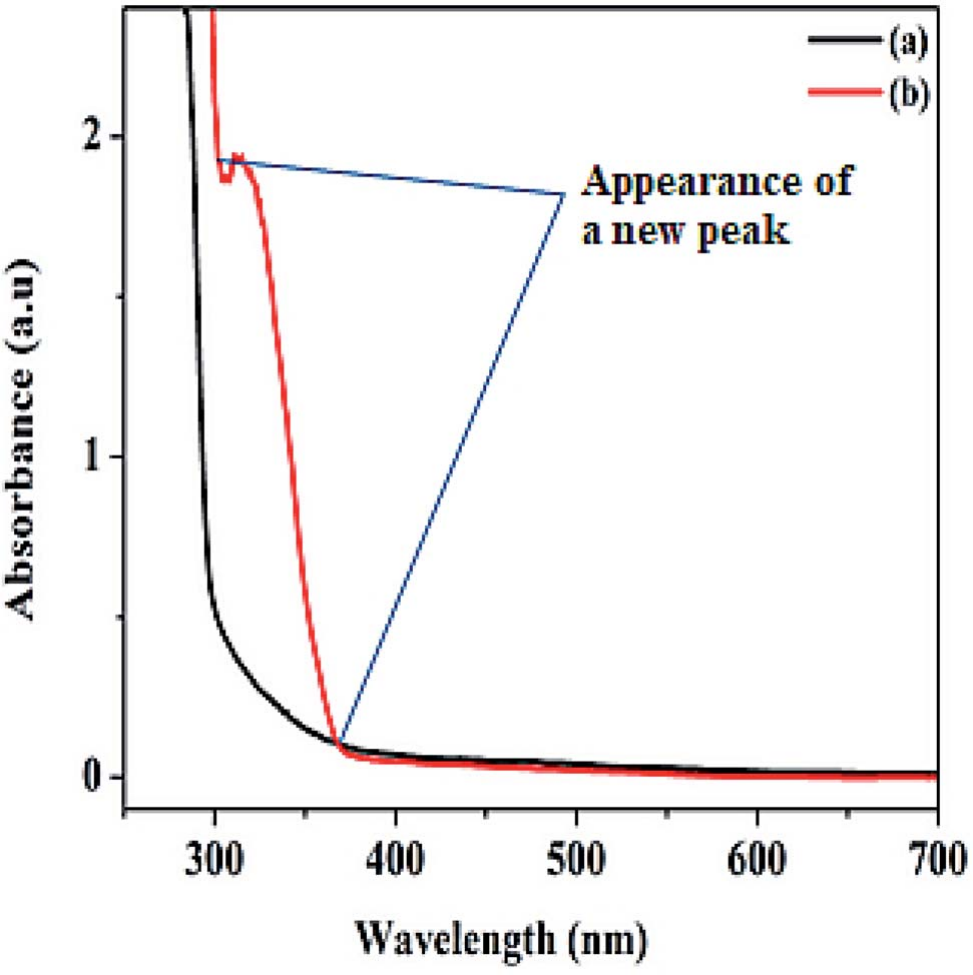
UV-vis spectra of (a) acetophenone substrate before catalysis, and (b) 1-phenyl ethanol after the product is formed.

The hydrogen nuclear magnetic resonance ^1^H NMR spectroscopy result shown in Fig. S2a–d† confirmed the molecular structure of our aromatic product. The spectra revealed a doublet at 1.47 ppm integrated for three protons, corresponding to methyl protons as expected since the methyl protons coupled to a proton at stereogenic carbon. In addition, the quartet multiplicity and integration of one proton on the stereogenic carbon at 4.86 ppm depicted the formation of 1-phenyl ethanol.^46^ Also, no substrate carbonyl carbon was seen at 7.30 ppm of 1-phenyl ethanol. The UV-Vis spectra recorded on a Shimadzu UV-1800 in a 3 mL quartz cuvette for both product formed and the substrate were performed within 250 and 700 nm range. As shown from the spectra (Fig. 5), two different characteristic absorption bands are displayed for the substrate and the product. The spectrum of 1-phenyl ethanol revealed a strong absorption peak between 299 and 367 nm, which may be ascribed to the formation of 1-phenyl ethanol in the electronic region of π → π* transition. The chromatogram of the identified product performed by the gas chromatography-mass spectrometry GC-MS equipment is also presented in Fig. S3.† The detail regarding the product extraction process before instrumental analysis can be found in the ESI.†

#### 3.2.1. Comparison test of HPMC solution with water

**Fig. 6:**
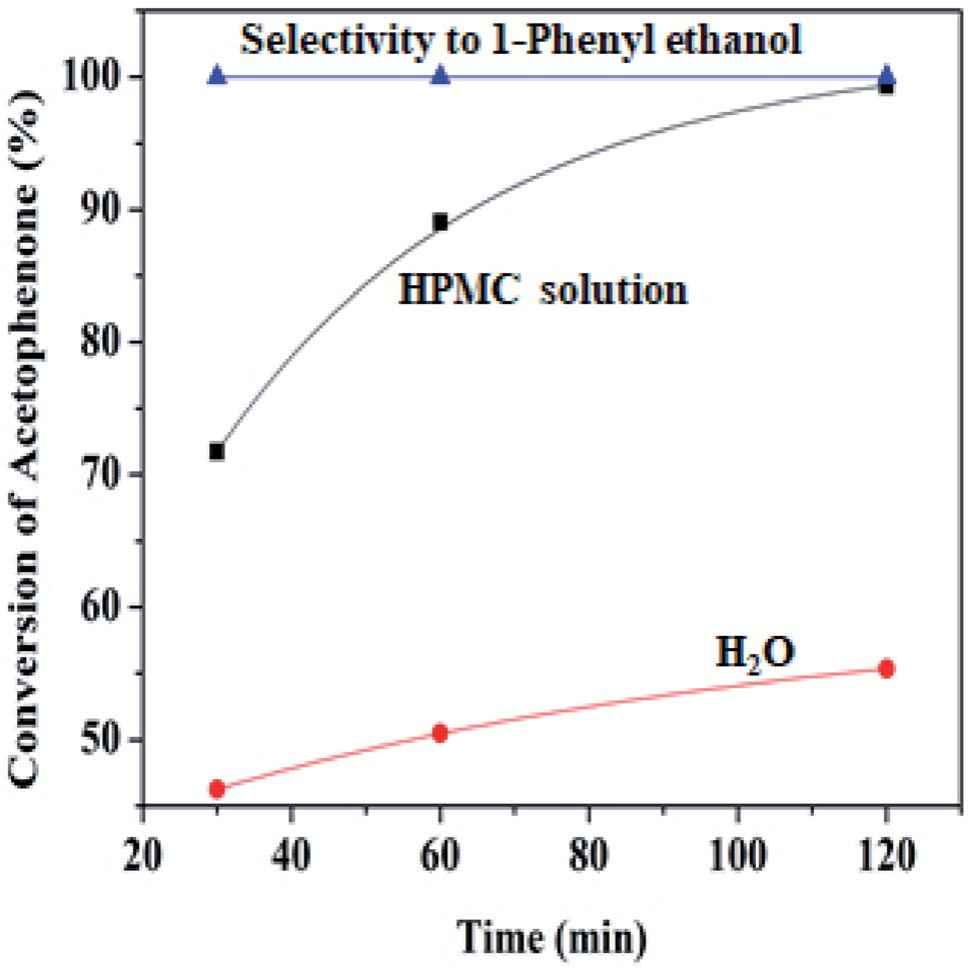
Percentage conversion comparison of HPMC modified water and unmodified water (6.00 mL) Pd@SiO_2_ (0.22 g), NaBH_4_ (1.00 g), acetophenone (21.60 mmol), decane (0.5 mmol), 80 °C.

A comparison test reaction was performed with time to investigate the efficiency of HPMC solution and water while using the same parameters and reaction conditions (0.22 g of Pd@SiO_2_, 80 °C, 21.60 mmol of the substrate, 1.00 g of redox agent, 6.00 mL of solvent, 0.5 mmol of decane). The result is illustrated in Fig. 6, and it shows that the conversion obtained in HPMC solution has a more significant influence upon acetophenone conversion to 1-phenyl ethanol. This more excellent performance is due to increased intermolecular hydrogen bonding generated.^47^ Although both mediums displayed a 100% total selectivity towards 1-phenyl ethanol, after 2 h, the highest conversion recorded in water was 55.32%, while 99.34% was observed for reaction in HPMC water. Therefore, HPMC solution was preferred above water to investigate other parameters.

At lower temperatures, HPMC displays liquid-like attributes that congeal upon an increase in temperature, where it potentially controls the structural and rheological pattern of the mixture.^48^ The hydroxypropyl group of the HPMC interacts with the molecule of water to form hydrogen bonds, while around the hydrophobic region (methyl groups), an enclosure of orderly arranged water in the form of ‘‘cages’’ is formed at a lower temperature. When the temperature increases, the system gains energy, and the hydrogen bonds break free from their enclosure while the clusters of hydrophobic groups are likewise released. This resulted in the formation of an interconnected network of viscoelastic thermal gel, which enhances higher interaction and stability with the reaction medium. Viscosity has been reported to improve stability within the colloidal dispersion by retarding the collapsibility of droplets and aggregation of particles.^11^ Hence, this behavioral pattern in water makes HPMC a biopolymer of special interest for drug formulation release.

#### 3.2.2. Effect of catalyst amount

**Fig. 7:**
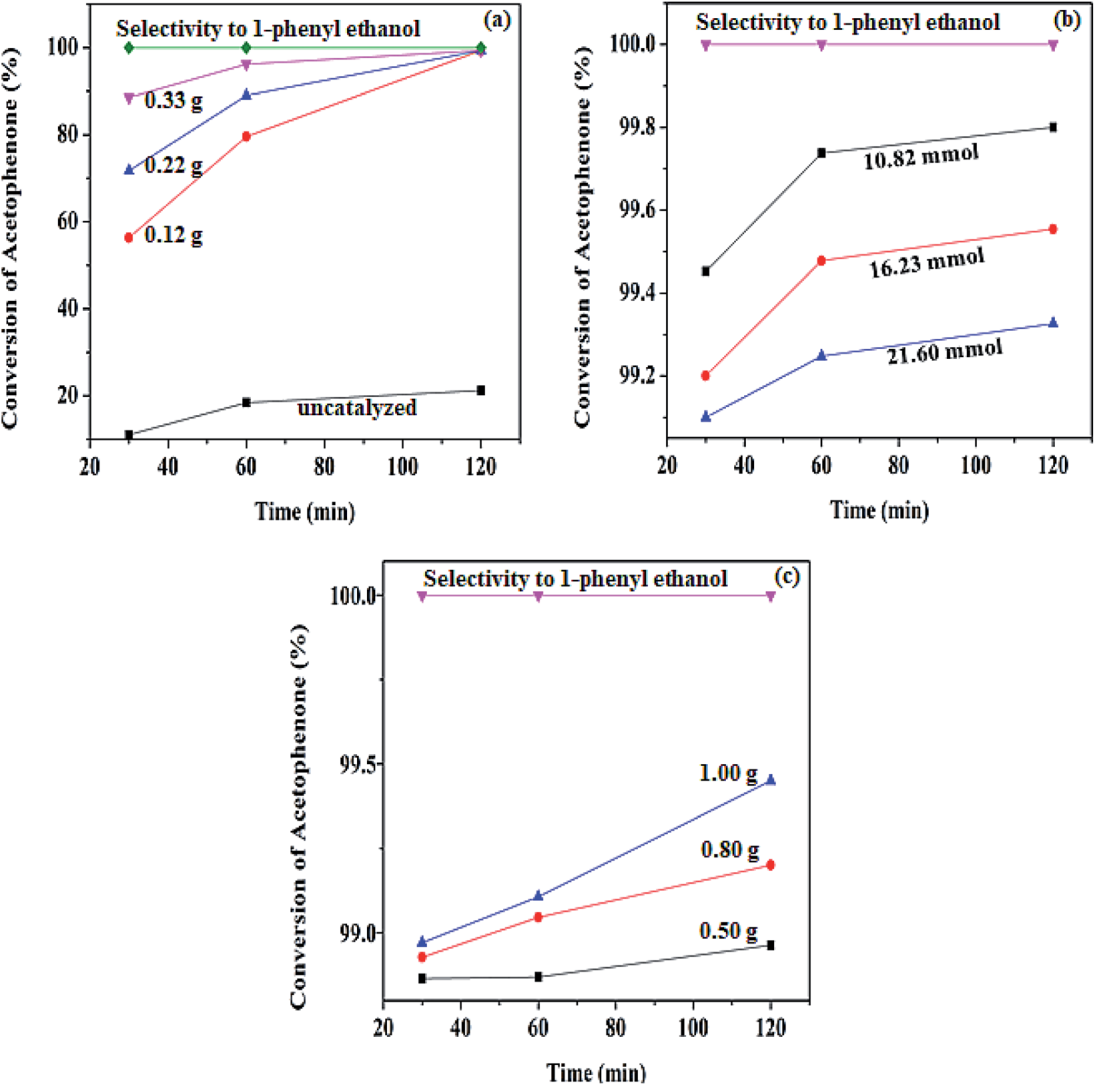
Different plots revealing the variations at 80 °C of (a) catalyst amount variation, (b) substrate variation, (c) borohydride variation obtained, using BH_4_^−^ (1.00 g), HPMC solution (6.00 mL), decane (0.5 mmol).

One of the crucial parameters in catalytic transformation is the catalyst amount because it enhances substrate conversion, selectivity, and product yield or formation, and it reveals reaction optimization. The different catalyst amounts were investigated using three amounts from 0.12 to 0.33 g while other parameters (21.60 mmol acetophenone, 1.00 g BH_4_^−^, 0.5 mmol decane, 6.00 mL HPMC solution) remained constant. The result is shown in Fig. 7a. All three variations showed a progressive conversion increase as the time increased, with a maximum conversion of 99.34% after 120 min at 80 °C. This result is due to an increase in the number of active sites of the catalyst as the reaction is taking place on the catalyst's surface. Initially, both BH_4_^−^ and the substrate are adsorbed on the catalyst surface. As the reaction proceeds, the carbonyl group of the acetophenone is activated by the catalyst to bond with the nucleophile of the BH_4_^−^. Then, the next step is the interaction of BH_4_^−^ and the carbonyl carbon atom on the catalyst's surface, thereby enhancing its electrophilicity.

Hence, the protons (H^+^) or electrophile released from the HPMC solution attacks the oxygen generated from the carbonyl group leading to the formation of 1-phenyl ethanol.^41^ The process is completed by the product's desorption from the catalyst surface, where a weak covalent bond initially bonded it. Therefore, the process continues in repetitive substrate adsorption and product desorption pattern. The hydrogenation process increases when the active sites are in abundance, while the reaction only ceases when the hydride ions produced by BH_4_^−^ are used up.^49^ The same conversion trend is seen in (Figure ESI) Fig. S4a and S5a† at different temperatures.

However, an increase in the conversion as the catalyst increases was observed, and the result is present in the ESI table (Table S1†) and to get the best optimum value for the catalyst loading, the turnover number (TONs) of the catalyst amount were calculated. From the calculation of the number TON (number of mole of substrate consumed/mole of catalyst),^50^ the lowest catalyst amount has the highest turnover number. The values obtained are; 3.679 × 10^3^, 2.009 × 10^3^, 1.335 × 10^3^ mol for 0.12, 0.22, and 0.33 g respectively. Moreover, to know the effect of hydrogen supply and the contribution of BH_4_^−^ used in the reaction, hydrogenation was carried out in the catalyst's absence. The result showed up to 21.21% conversion of acetophenone at 80 °C, while the reaction did not proceed in the absence of both the catalyst and the supply of hydrogen. This indicates that the reaction is mainly a function of the catalyst.

#### 3.2.3. Effect of substrate amount

The substrate variation was observed using three amounts from 10.82, 16.23, to 21.60 mmol and is represented in Fig. 7b. The result showed an increase in conversion trend as the time increased, with the highest conversion of 99.80% recorded in 120 min at 80 °C using a constant parameter of 0.22 g of Pd@SiO_2_, 1.00 g of BH_4_^−^, 0.5 mmol decane, and 6.00 mL HPMC solution. The conversion trends as the substrate amount increased is >99.80%, >99.55, and >99.32% for 10.82 mmol, 16.23, and 21.60 mmol, respectively, after 120 min. Therefore, 10.82 mmol of the substrates illustrated the best condition for the effect of substrate amount. However, it is worth noting that the increase in conversion with the decrease in substrate amount may be due to the higher solubility of the substrate obtained in HPMC solution at a minimum substrate amount. Organic substrate dissolution and reactivity to form products are enhanced in HPMC solution. In addition, these patterns could also result from the saturation of the substrate upon the catalyst surface. Other plots obtained from the data acquired for the substrate variation at different temperatures are shown in Fig. S4b and S5b.† ESI Table (ST) S1† also presented the data obtained for the different temperatures at the same amount of catalyst and the substrate.

#### 3.2.4. Effect of borohydride amount

The investigation carried out on the effect of BH_4_^−^ was performed using three different parameters as well, while other reagents were kept at a constant amount (0.22 g of Pd@SiO_2_ 21.60 mmol acetophenone, 0.5 mmol decane, and 6.00 mL HPMC solution). As shown in Fig. 7c, the result revealed an increase in the percentage conversion trend as the BH_4_^−^ increases. This indicates an increase in the generation of the nucleophile, which is attacked by the electrophile from the water leading to an increase in the desorption of the product formed from the surface of the catalysts. The pattern of increase in the conversion as the hydrogen donation increases was also observed at other temperatures. This is illustrated in Fig. S4c and S5c.†

#### 3.2.5. Effect of reaction temperature

**Fig. 8:**
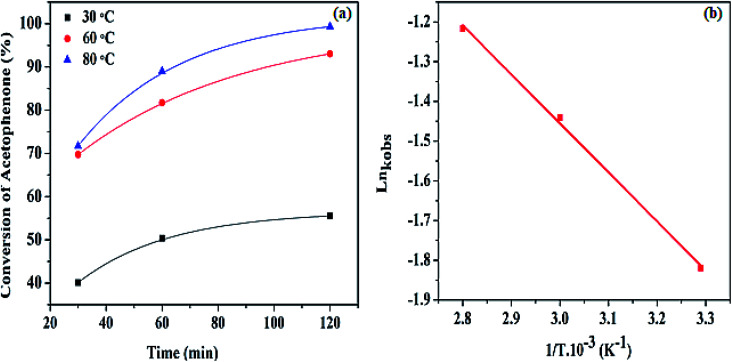
(a) Percentage conversion of acetophenone at different temperatures, (b) the Arrhenius plot of Ln *k*_obs_ against 1/*T* at various temperatures.

Fundamentally, an increase in temperature is known to favor chemical reactions, however, the effect of temperature investigation on the transfer hydrogenation of acetophenone was still probed using three different temperatures of 30, 60, and 80 °C. This was investigated to obtain optimum temperature conditions. The amount of catalyst used and other reactant parameters were kept constant while changing the temperature. The graphical plot is illustrated in Fig. 8a. At each temperature variation, selectivity of 100% toward 1-phenyl ethanol was achieved with an overall highest percentage of 65.62, 96.22, and 99.80% at 30, 60, and 80 °C respectively, using the minimum substrate amount (10.82 mmol). An increase in the catalytic conversion was obtained as the temperature increased. This may as a result of modulated enlargement in molecular diffusion at a higher temperature, leading to an improved conversion rate and enhanced productivity.^51^ Moreover, increasing temperature creates a higher interacting environment for both the hydrophobic and hydroxyl (hydrophilic) counterparts in thermal gel such as HPMC.^48^ This is also demonstrated in the observed rate constant *k*_obs_ computed, in the order of 0.16 min^−1^ < 0.24 min^−1^ < 0.29 min^−1^ at 30, 60, and 80 °C respectively. The graph of *k*_obs_ against the temperature is revealed in Fig. S6.†

The observed activation energy for the Pd@SiO_2_ catalyst was computed from the graph of Ln *k*_obs_ against (1/*T*) as presented in Fig. 8b. To the best of our knowledge, there are only a few reports on the activation energy for acetophenone hydrogenation. The estimated value obtained was discovered to be 19.05 kJ mol^−1^. This is an indication of a typical catalytically driven chemical reaction.

### 3.3 Turnover frequency of Pd@SiO_2_ catalyst

The catalyst's turnover frequency (TOF) is one of the crucial tools in estimating or probing the activity of a catalyst.^52^ It is a valuable assessment used to compare the activity of catalysts having the same characteristic domain.^50^ The TOF can be expressed as the number of converted moles of acetophenone (substrate) divided by the number of loaded catalysts amounts and reaction time. Table 1 presents the TOF calculated for the conversion of acetophenone at different temperatures. The result obtained showed a consistent increase in TOF as the temperature increases. We can say that increase in temperature significantly contributes to and influences the activity of the Pd@SiO_2_ catalyst. In addition, product formation also improved at higher temperatures.

**Table 1:** Data showing the increasing order of the TOF obtained at different temperatures^a^

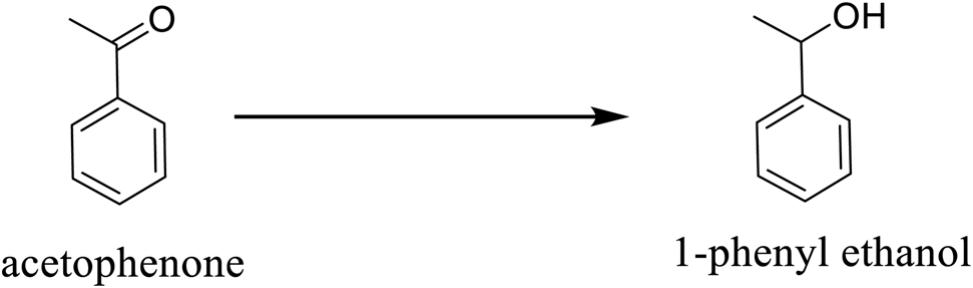	
Temperature (°C)	TOF (min^−1^)
30	368.31
60	585.57
80	625.29

a Conditions: Pd@SiO_2_ (0.22 g), BH_4_^−^ (1.00 g), HPMC solution (6.00 mL), acetophenone (21.60 mmol), decane (0.5 mmol), 80 °C.

### 3.4 Catalyst recyclability and stability

**Fig. 9:**
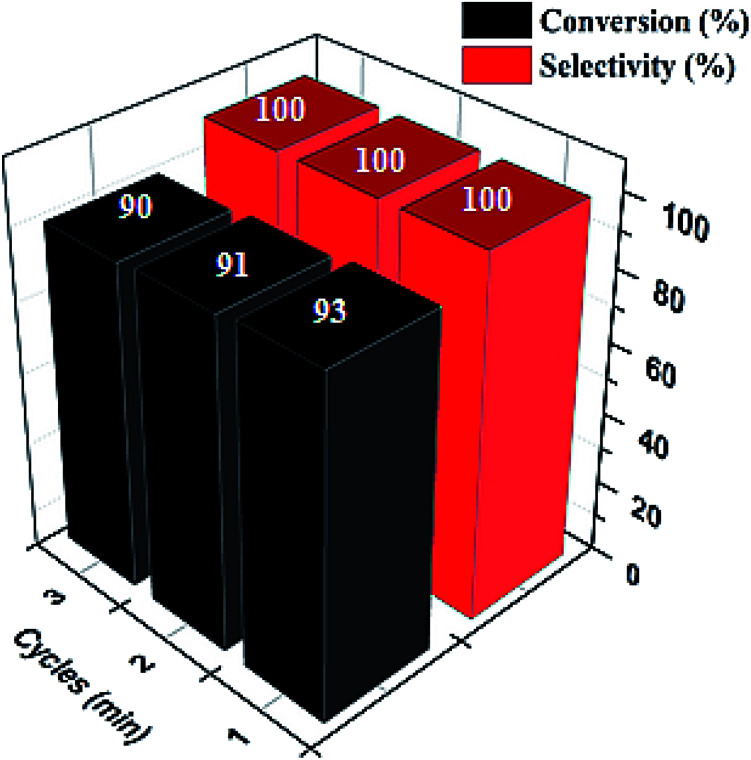
Recyclability plot of acetophenone conversion to 1-phenyl ethanol (at 80 °C, 0.22 g catalyst, 21.60 mmol of acetophenone, 1.00 g BH_4_^−^ and 0.5 mmol decane).

Ultimately, a critical aspect of heterogeneity in catalysis is the ability to recover and reuse catalysts with an insignificant change in their activities. This attribute confirms their stability in the reaction media. Pd@SiO_2_ catalyst's stability was investigated over a period of 3-cycles in the reaction medium, as displayed in Fig. 9. The result showed that the catalyst had no significant deactivation or reduction in activity as the number of cycles increased. The selectivity toward 1-phenyl ethanol remained at 100% for the 3-cycles, while the conversion obtained at the third cycle was 90%. This result represents a good characteristic of Pd@SiO_2_ catalyst stability.

Furthermore, ICP-OES measurements also revealed only negligible Pd (0.05 mg L^−1^) was available within the reaction medium at the end.

### 3.5 Transfer hydrogenation with other ketones

In addition, the hydrogenation reaction was carried out with 5 varieties of ketone substrates which are propiophenone, 3-pentanone, 2-pentanone, cyclohexanone, and fenchone. An excellent qualitative conversion was achieved for all the substrates, as shown in Table 2, from entries 2 to 6.

**Table 2:** Reduction of other ketone substrates by Pd@SiO_2_^a^

Entry	Substrate	Product	Time (min)	Conversion (%)
1	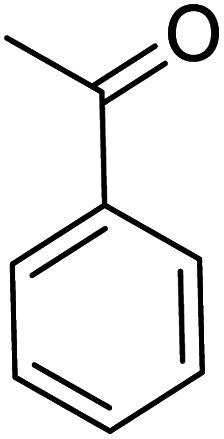	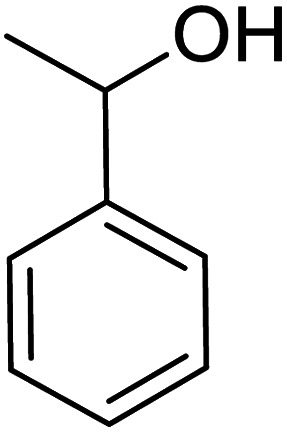	120	96.21
2	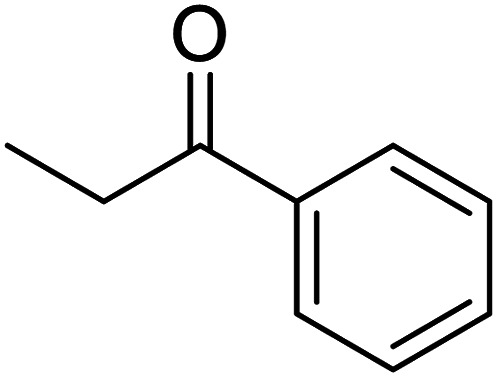	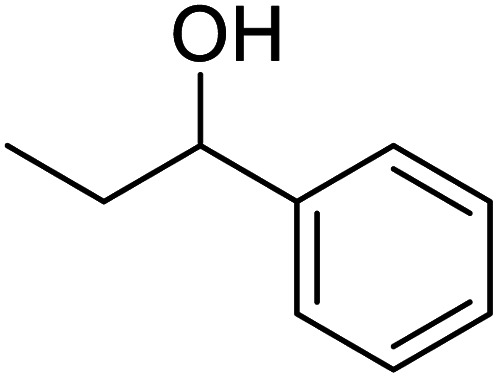	120	94.64
3	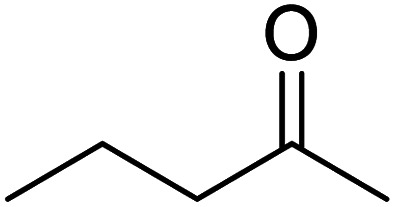	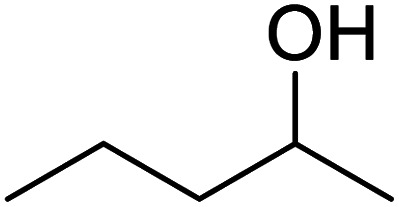	120	94.38
4	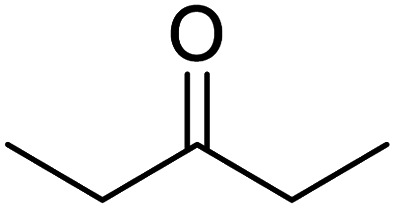	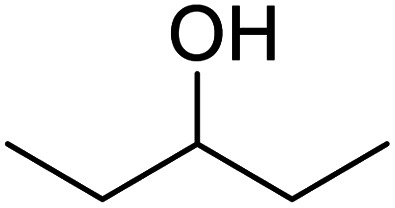	120	98.49
5	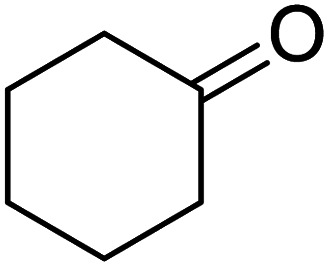	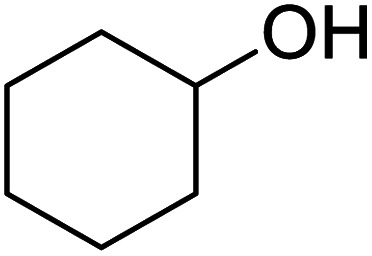	120	96.48
6	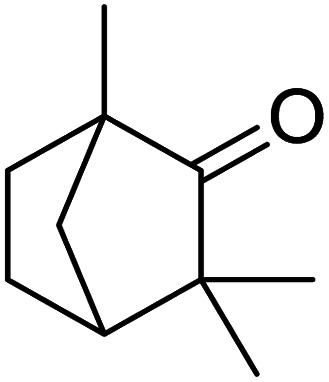	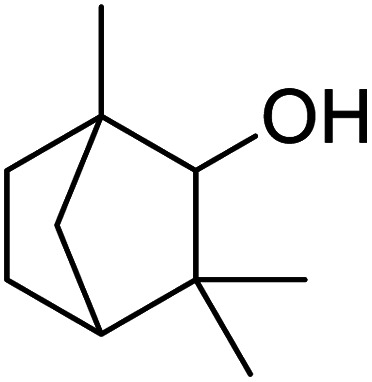	120	95.97

a Condition: 21.60 mmol of substrate in 6.00 mL HPMC solution, and 0.5 mmol decane at 80 °C.

The NMR analysis obtained for the structural product identification is shown in Fig. S7a and b, S9a and b, S11a and b, S13a and b, and S15a and b.† Further product confirmation was carried out through GC-MS analysis, and it revealed the various products generated after the hydrogenation. The results obtained are shown in Fig. S8, S10, S12, S14, and S16.†

### 3.6 Comparative study of Pd@SiO_2_ with other Pd based catalyst

A comparison of Pd@SiO_2_ activity for the hydrogenation of acetophenone was made with the previous reports to support the effectiveness of the catalyst. This is summarized in Table 3. From the summary, the conversion and selectivity obtained for using Pd@SiO_2_ catalysts are impressively comparable to the reported values in the table. Also, the time taken to produce 1-phenyl ethanol depicted an impressive performance. The parameters obtained for TOF calculation also expressed the efficiency and capability of Pd@SiO_2_ in the hydrogenation reaction regarding other values in the literature. Moreover, the energy of activation obtained in this report which is lower than those in previous reports presented the needed energy that can substantially yield the production of 1-phenyl ethanol.

**Table 3:** Comparison of Pd@SiO_2_ catalysts activity with Pd-based catalysts previous reports towards hydrogenation of ketone^a^

Catalyst	Hydrogen source	Conversion (%)	Selectivity (%)	Temp (°C)	Time (min)	*E*_a_ (kJ mol^−1^)	Product	TOF (min^−1^)	Ref.
PdNPs	NaBH_4_	99.8	100	28.0	6	—	Isopropanol	—	29
Pd/SA	Pressurized H_2_	95	55	60.0	60	—	Ethylbenzene	—	30
Pd/C	Pressurized H_2_	96.0	96	39.9	180	—	1-Phenyl ethanol	—	53
Pd/N-VGCF^c^	ATM H_2_	77	31	70.0	300	—	(*R*)-1-Phenyl ethanol	116.7	42
Pd/N-VGCF^b^	ATM H_2_	76	33	70.0	300	—	(*R*)-1-Phenyl ethanol	83.3	42
Pd@Ph-POP	Pressurized H_2_	100	100	60.0	60	38.00	Ethylbenzene	5.42	43
Pd@Al_2_O_3_	Pressurized H_2_	75	3	60.0	60	124.00	Ethylbenzene	3.80	43
Pd-RuEnCat	Pressurized H_2_	97	71	80.0	240	61.90	1-Phenyl ethanol	—	25
Pd@SiO_2_	NaBH_4_	99.8	100	80.0	30	19.05	1-Phenyl ethanol	625.29	TW

a Temp = temperature, Ref = reference, TW = this work.

b Pd catalysts pre-reduced at 100 °C for 30 min

c Pd catalysts pre-reduced at 200 °C for 120 min

## 4. Conclusion

The Pd@SiO_2_ catalyst was successfully synthesized, and the solvent used was an advantage for the effective reduction of acetophenone. This is due to silanol groups occupying the catalyst's surface, making the adsorption of acetophenone and hydrogen easy and quick. The catalytic reduction of acetophenone in HPMC solution using BH_4_^−^ as a hydrogen source is demonstrated as alternative means of hydrogenation because it is attractive, efficient, effective, and eco-friendly. The investigation revealed the HPMC solution as a promising channel for the sustainable catalytic transformation of acetophenone. Moreover, an enabling reaction medium was generated through intermolecular interaction from the HPMC at a higher temperature. Also, the nucleophile generated through the BH_4_^−^ effectively reacted with the oxygen present in the carbonyl group before interacting with the protons (H^+^), which resulted in the formation of 1-phenyl ethanol.

The selectivity of 1-phenyl ethanol was maintained at 100%, while the highest conversion obtained was 99.80% without any unwanted product or by-product. The TOF values recorded for the Pd@SiO_2_ catalyst activity increased with increased temperature having the highest value of 625.29 min^−1^. Also, the activation energy was calculated to be 19.05 kJ mol^−1^. The catalyst activity was still maintained after 3-cycles, and only 0.05 mg L^−1^ of Pd@SiO_2_ catalyst was present in the reaction medium, which is a negligible amount.

## Conflicts of interest

There are no conflicts to declare.

## Supplementary Material

RA-012-D2RA02701A-s001
