# Supplementary material for: Transfer hydrogenation of ketone; an in situ approach toward an eco-friendly reduction
Source: RSC Adv. 2022 Jul 7;12(31):19890–900. doi: 10.1039/d2ra02701a (PMC9262422; doi:10.1039/d2ra02701a)
Supplement: RA-012-D2RA02701A-s001 [file RA-012-D2RA02701A-s001.pdf]

Electronic Supplementary Material (ESI) for RSC Advances.
This journal is © The Royal Society of Chemistry 2022

Transfer Hydrogenation of Ketone; An *In-situ* Approach toward an Eco-friendly Reduction

Oluwatayo Racheal Onisuru, Dele Peter Fapojuwo, Charles O. Osaghale, Oyekunle Azeez Alimi, and Reinout Meijboom

Department of Chemical Sciences, University of Johannesburg, P.O. Box 524, Auckland Park, Johannesburg 2006, South Africa.

Tel. +27 (0)728940293, Fax: +27 (0)11 559 2819

Corresponding Author* E-mail: rmeijboom@uj.ac.za (R.M.)

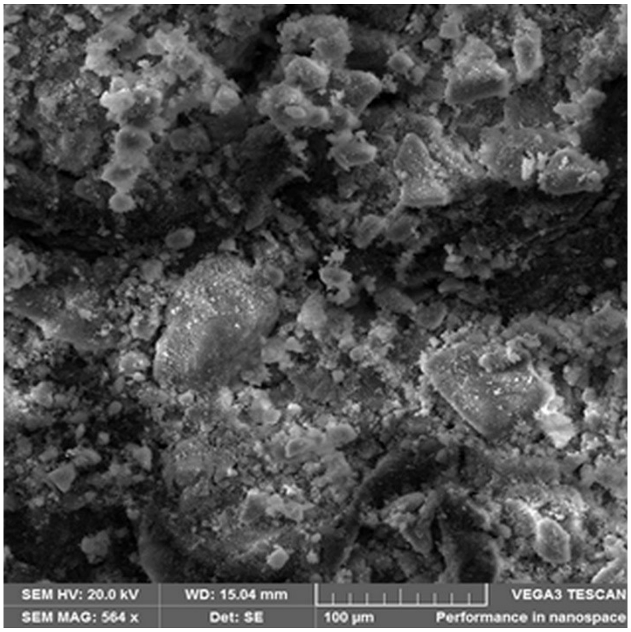

Fig. S1. Image of SEM obtained for Pd@SiO_2

$$Conversion\ (\%) = \frac{(Substrate_0 - Substrate_t) \times 100}{Substrate_0} \tag{1}$$

$$Selectivity\ (\%) = (\frac{moles\ of\ product}{moles\ of substrate_0}) \times 100 \tag{2}$$

Substrate$_0$ represents the initial mole of the substrate, and substrate$_t$ is the present mole of the substrate at the time t.

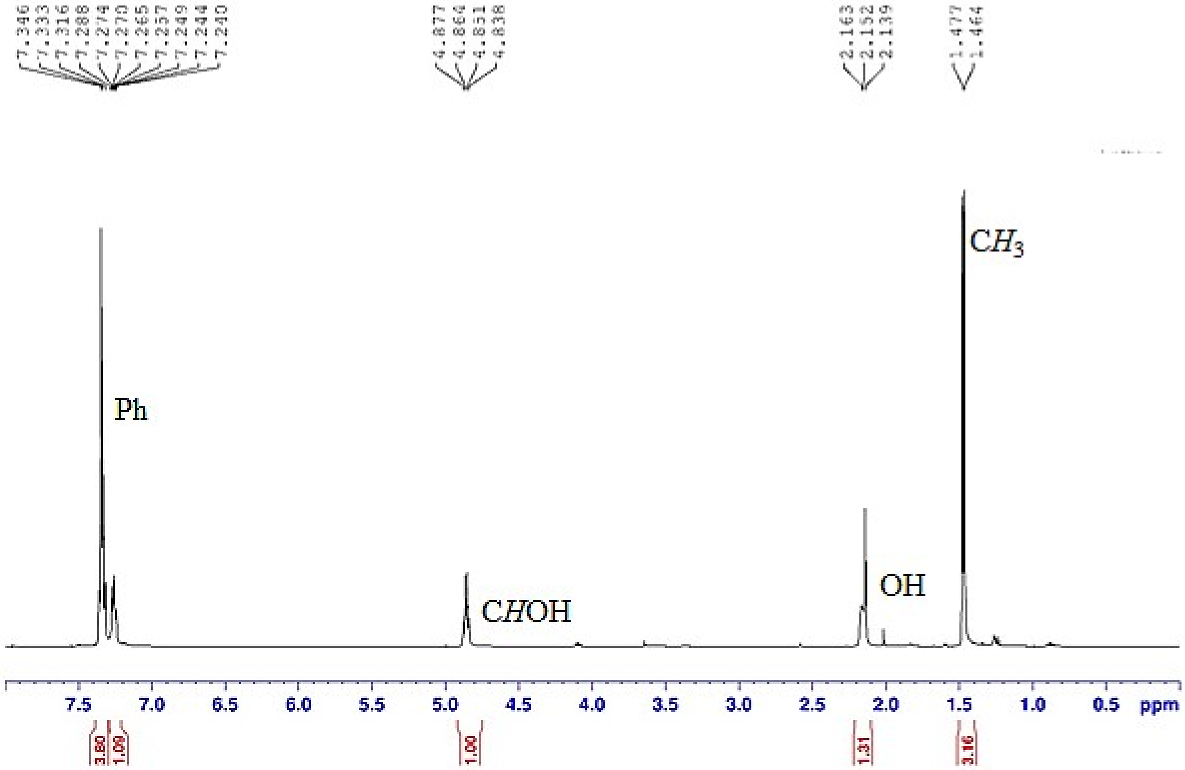

Fig. S2a. ^{1}H NMR Spectrum of the produced 1-Phenyl ethanol using Pd@SiO$_2$.

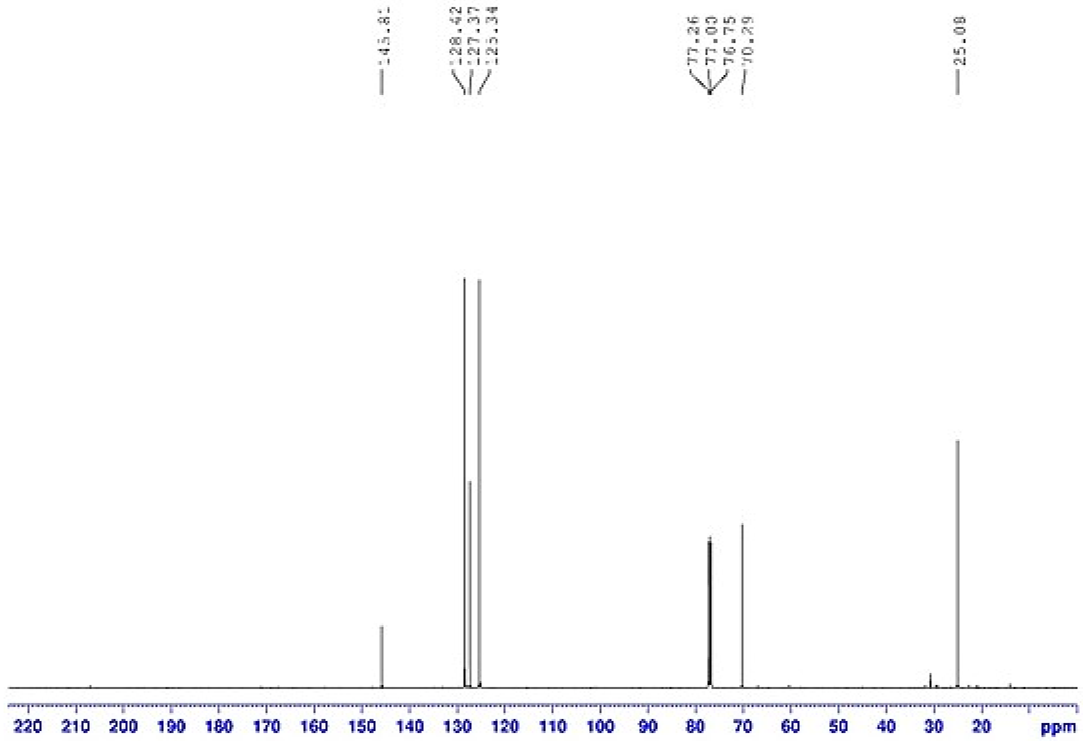

Fig. S2b. ^{13}C NMR Spectrum of the produced 1-Phenyl ethanol using Pd@SiO_2.

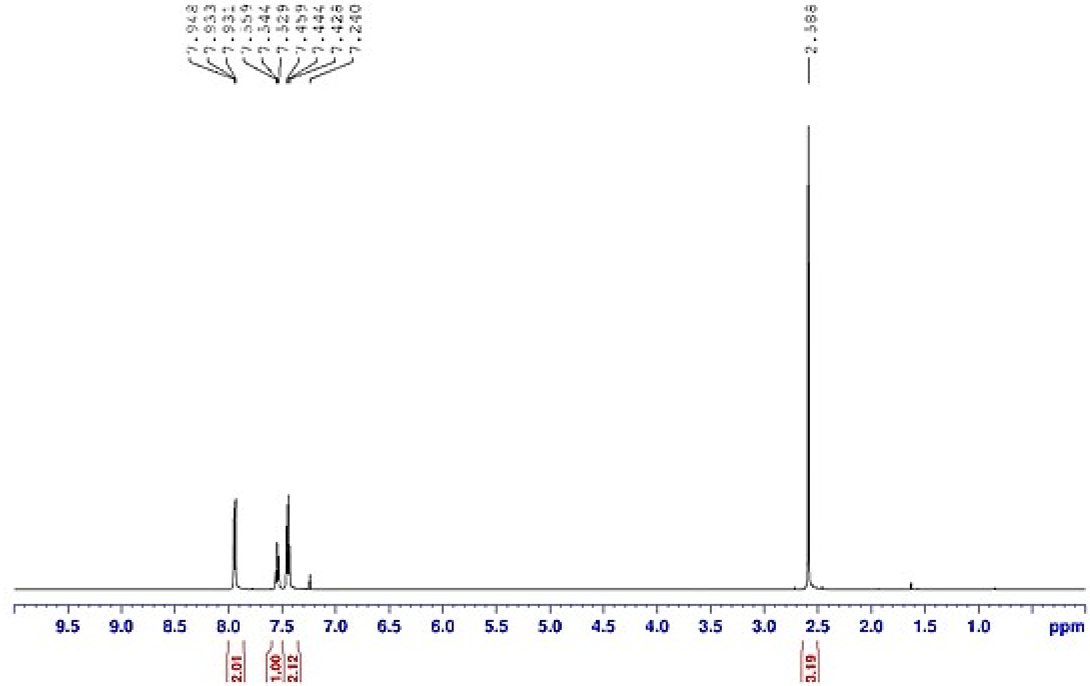

Fig. S2c. ^{1}H NMR Spectrum of the produced 1-Phenyl ethanol using Pd@SiO_2.

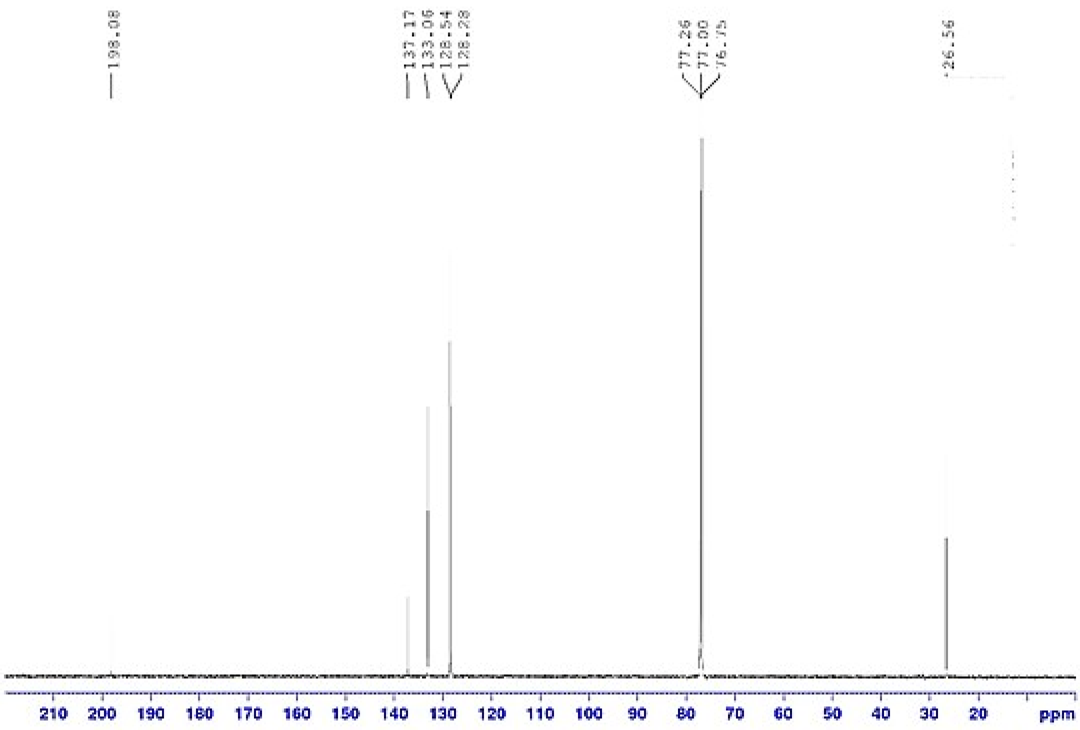

Fig. S2d. ^{13}C NMR Spectrum of the produced 1-Phenyl ethanol using Pd@SiO_2.

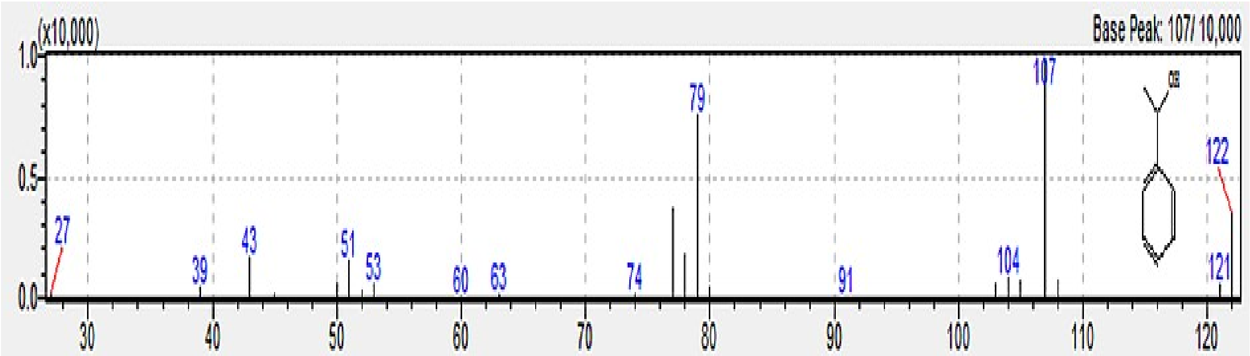

Fig. S3. Product chromatogram of the GC-MS analysis obtained for the hydrogenation of acetophenone at 80 °C after 30 min.

Generally from the literature ketone hydrogenation leads to the formation of one product. [1–6] Therefore, the extraction process on our product to remove all possible solvents was carried out using BUCHI rotavapor R-100 after the filteration of the organic layer. Product separation was done with ethyl acetate of which the organic layer was passed through a column of $MgSO_4$ dried.

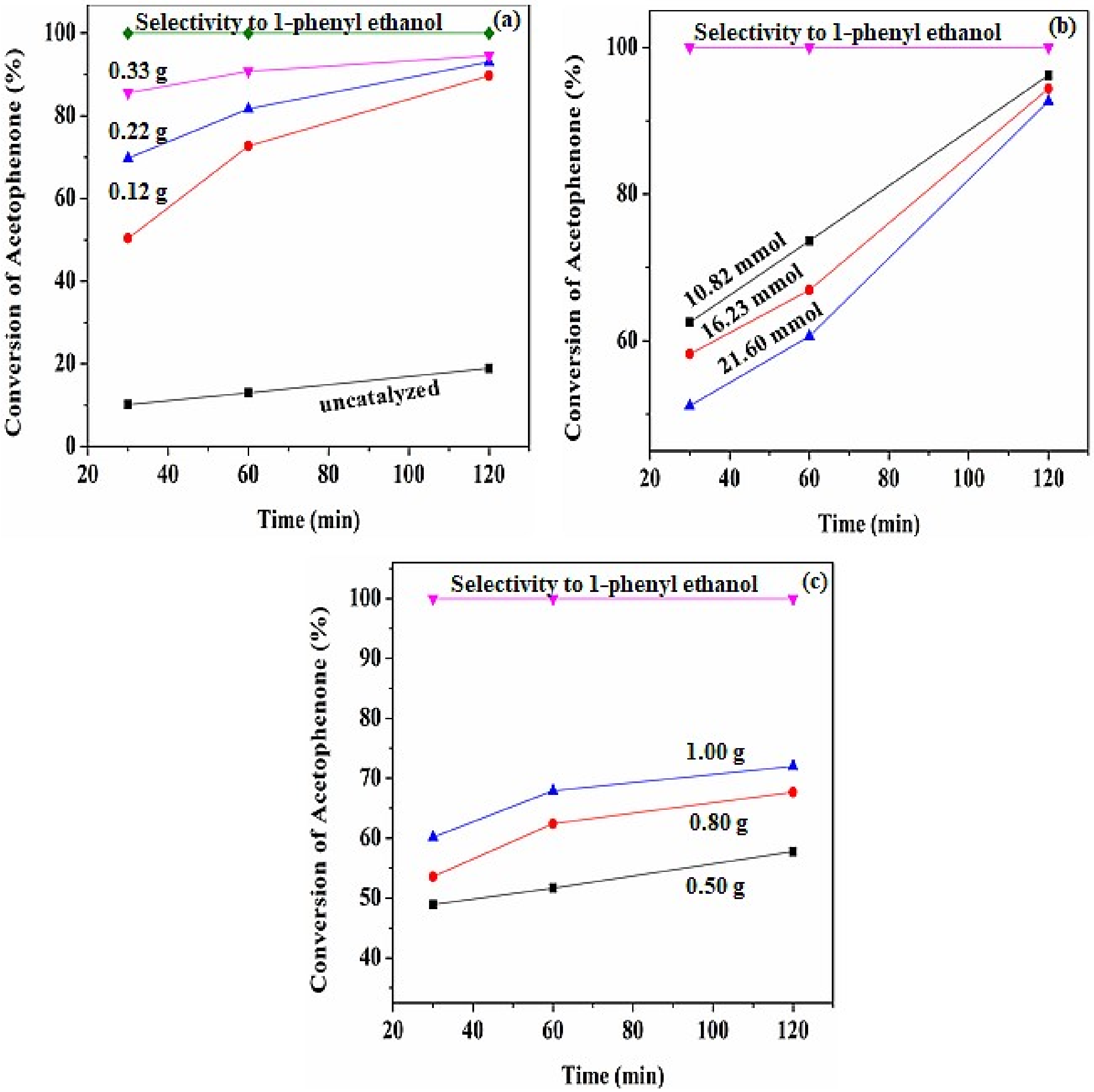

Fig. S4. Different plots revealing the variations at 60 °C of (a) catalyst amount variation, (b) substrate variation, (c) Borohydride variation obtained.

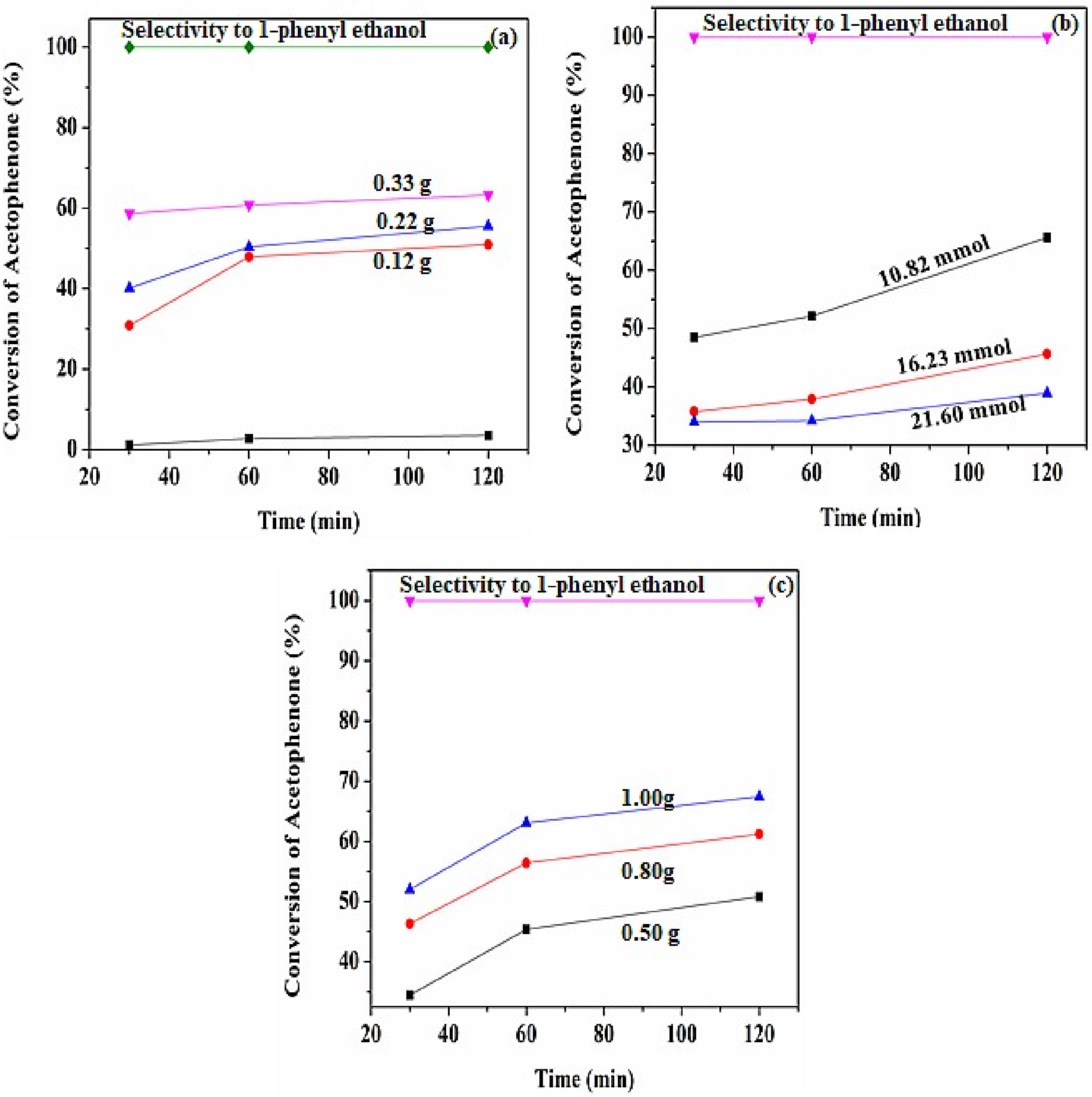

Fig. S5. Different plots revealing the variations at 30 °C of (a) catalyst amount variation, (b) substrate variation, (c) Borohydride variation obtained.

Supplementary Table (ST) 1

Data obtained for the conversion of acetophenone at different temperatures using the same amount of catalyst and the substrate.

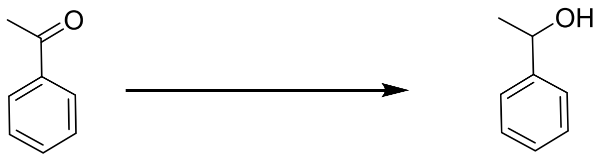

acetophenone 1-phenyl ethanol

Temperature (°C)	Conversion (%)		
	at 30 (min)	at 60 (min)	at 120 (min)
30	33.99	34.21	38.93
60	51.15	60.63	92.67
80	99.09	99.25	99.33

Condition: 21.60 mmol of the substrate, 1.00 g of BH_4^-, 0.22 g of Pd@SiO_2, 6.00 mL of HPMC solution decane (0.5 mmol), 80 °C.

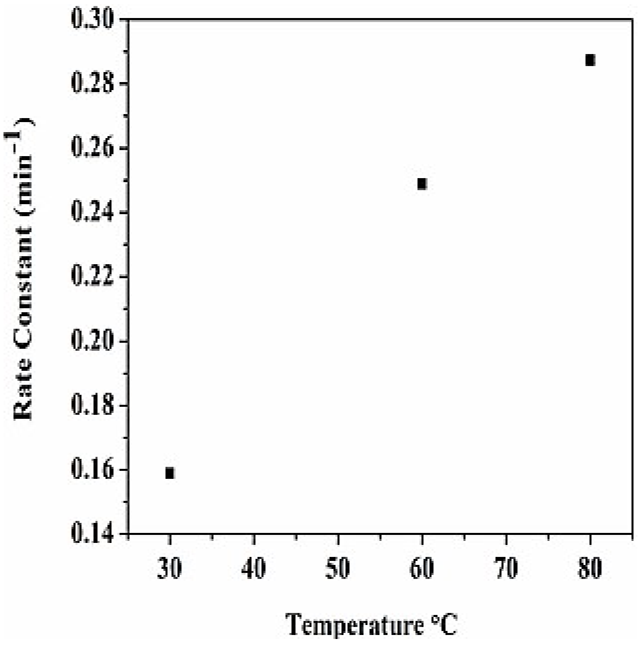

Fig. S6. Plot showing the dependence of the rate constant on the temperature.

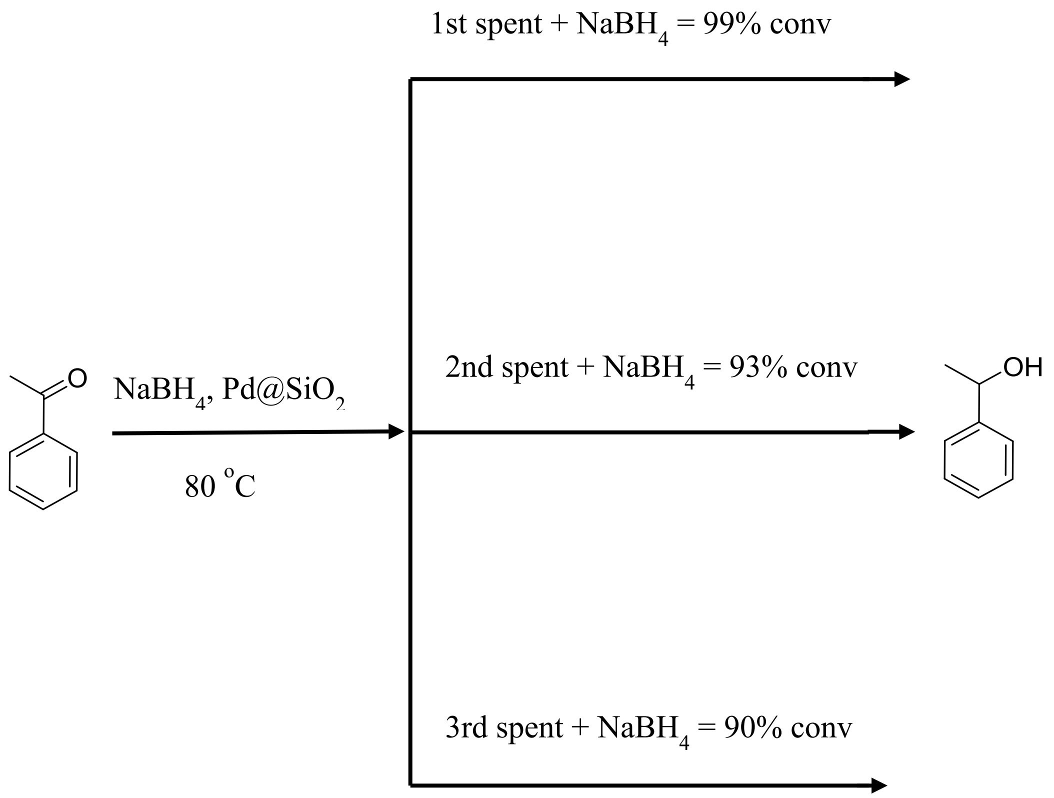

SShceme 1. Schematic representation of the recyclability of spent catalyst acetophenone conversion (conv) to 1-Phenyl ethanol (at 80 °C, 0.22 g catalyst, 21.60 mmol of acetophenone, 1.00 g BH_4^- and 0.5 mmol decane).

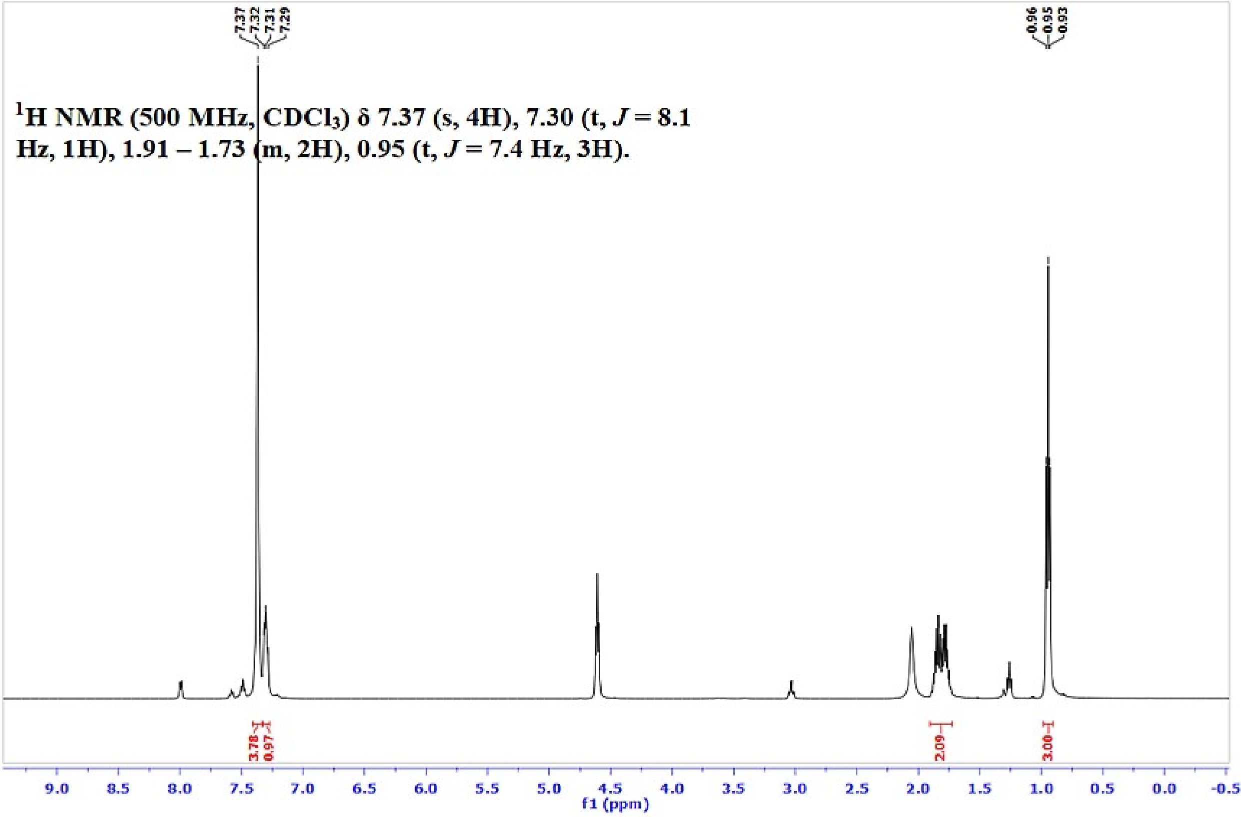

Fig. S7a. ^{1}H NMR Spectrum of the generated benzene methanol using Pd@SiO_2.

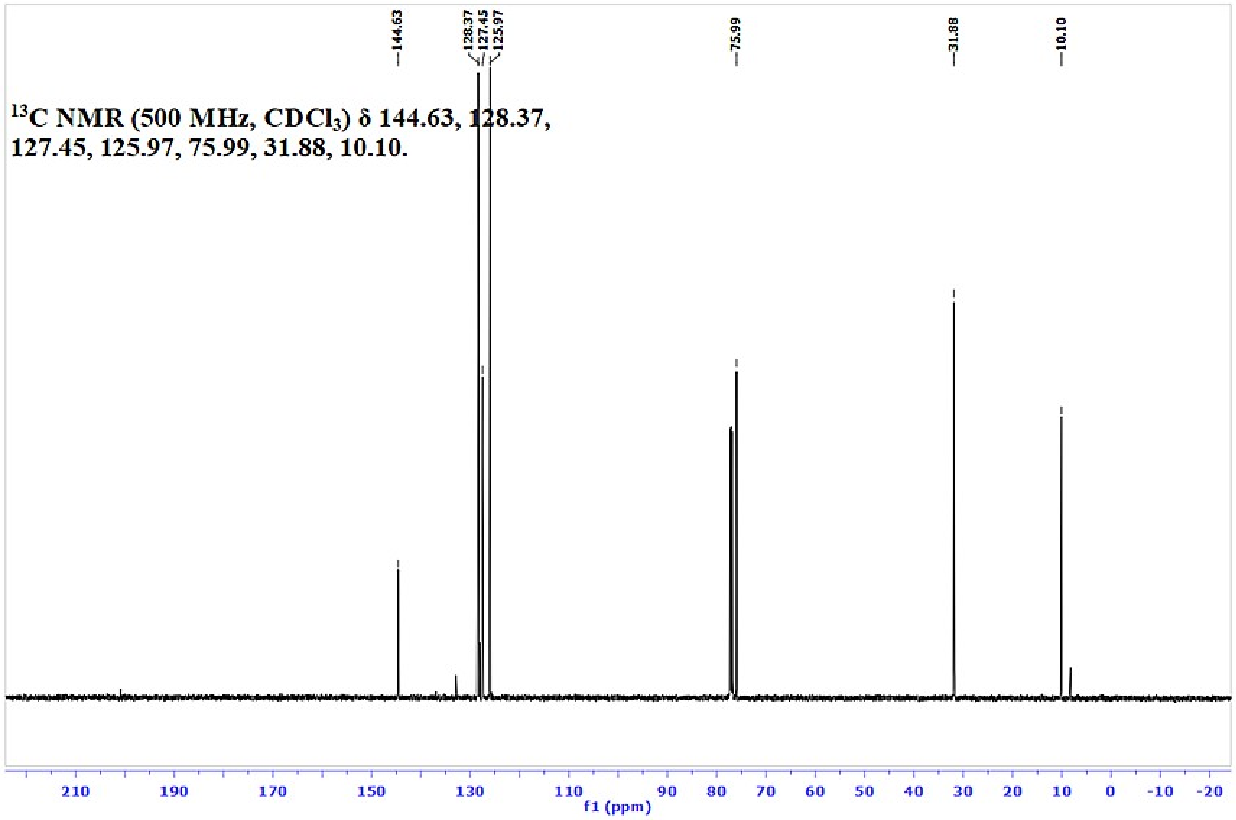

Fig. S7b. ^{13}C NMR Spectrum of the generated benzene methanol using Pd@SiO_2.

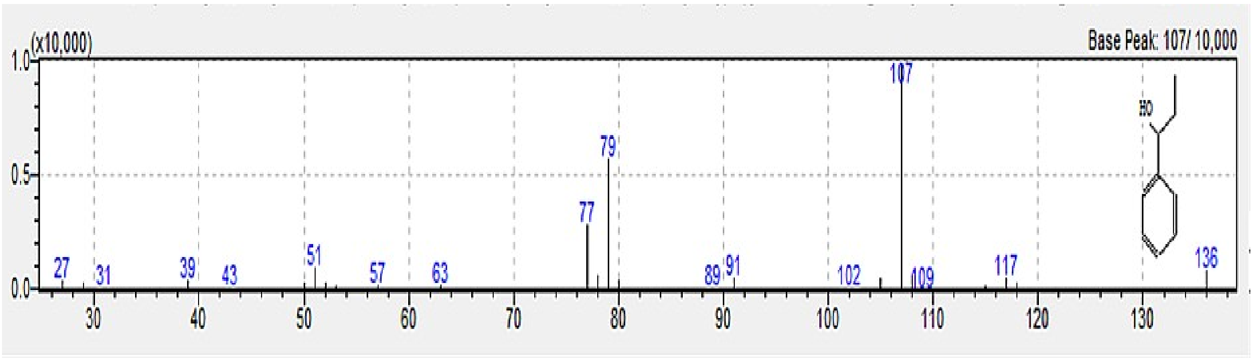

Fig. S8. Product chromatogram of the GC-MS analysis obtained for the hydrogenation of propiophenone at 80 °C.

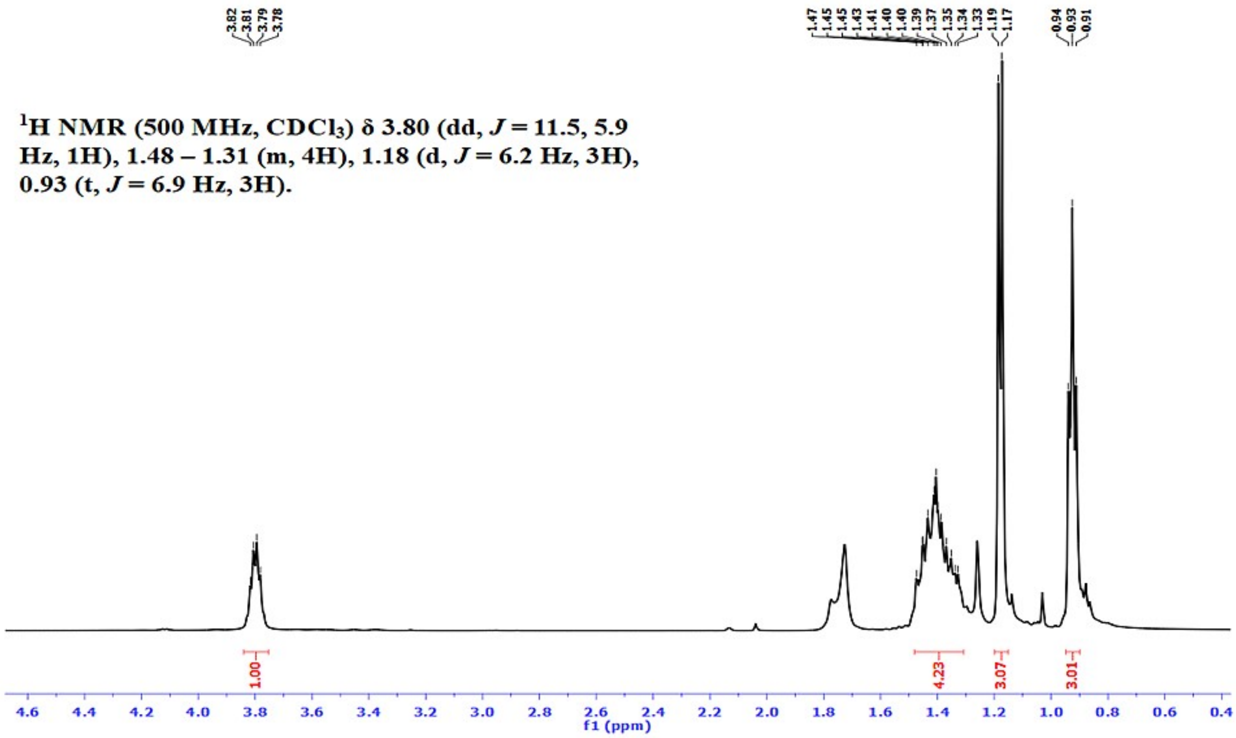

Fig. S9a. ^{1}H NMR Spectrum of the generated 2-Pentanol using Pd@SiO_2.

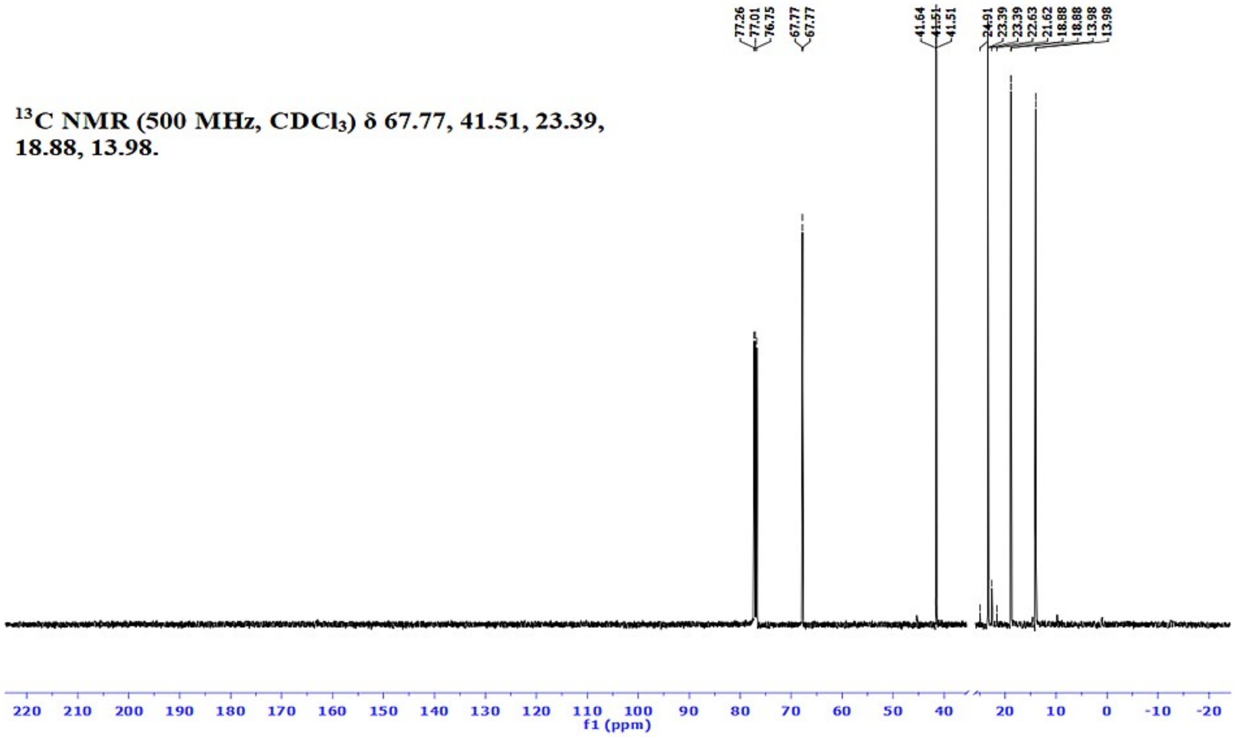

Fig. S9b. ^{13}C NMR Spectrum of the 2-Pentanol using Pd@SiO_2.

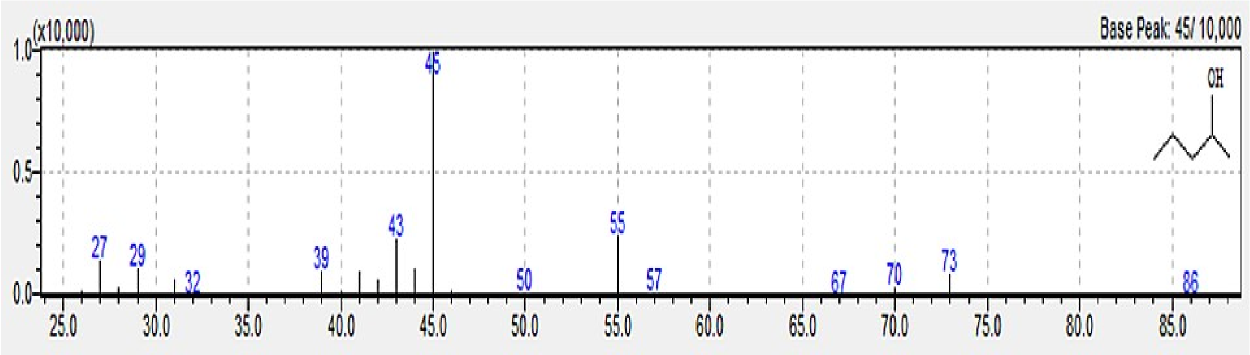

Fig. S10. Product chromatogram of the GC-MS analysis obtained for the hydrogenation of 2-Pentanone at 80 °C.

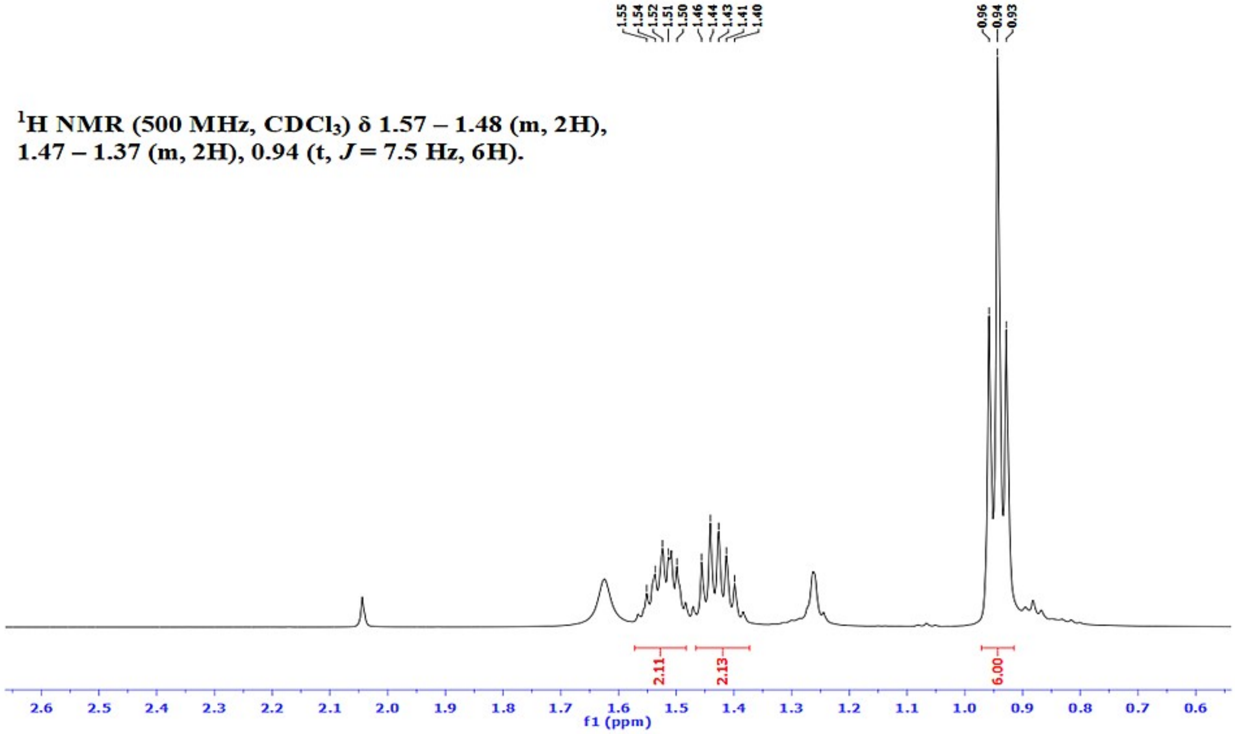

Fig. S11a. ^{1}H NMR Spectrum of the generated 3-Pentanol using $Pd@SiO_2$.

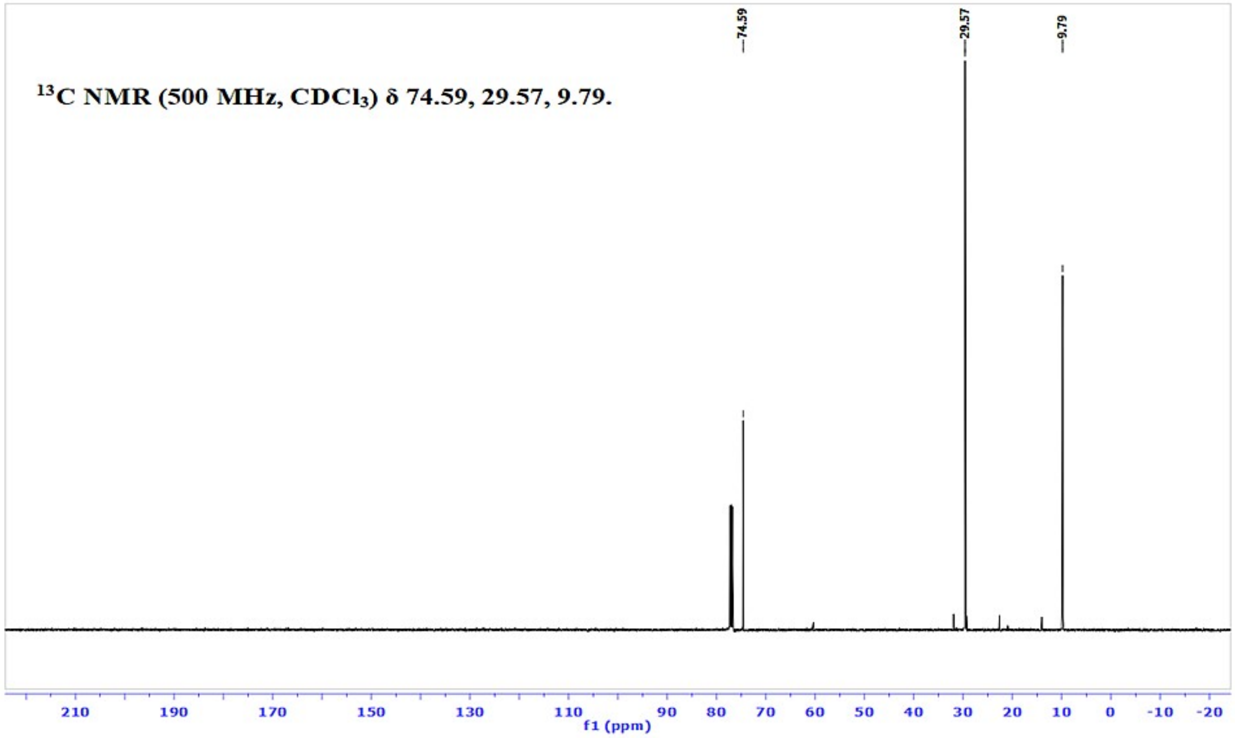

Fig. S11b. ^{13}C NMR Spectrum of the 3-Pentanol using Pd@SiO_2.

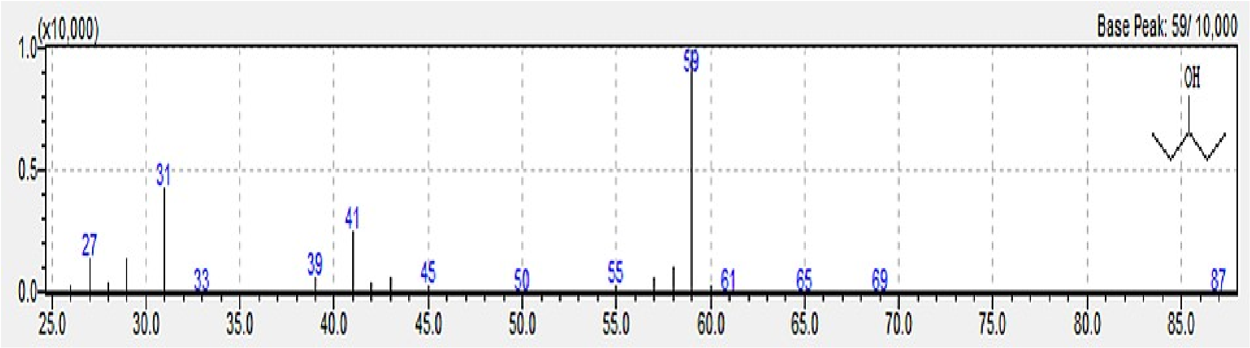

Fig. S12. Product chromatogram of the GC-MS analysis obtained for the hydrogenation of 3-Pentanone at 80 °C.

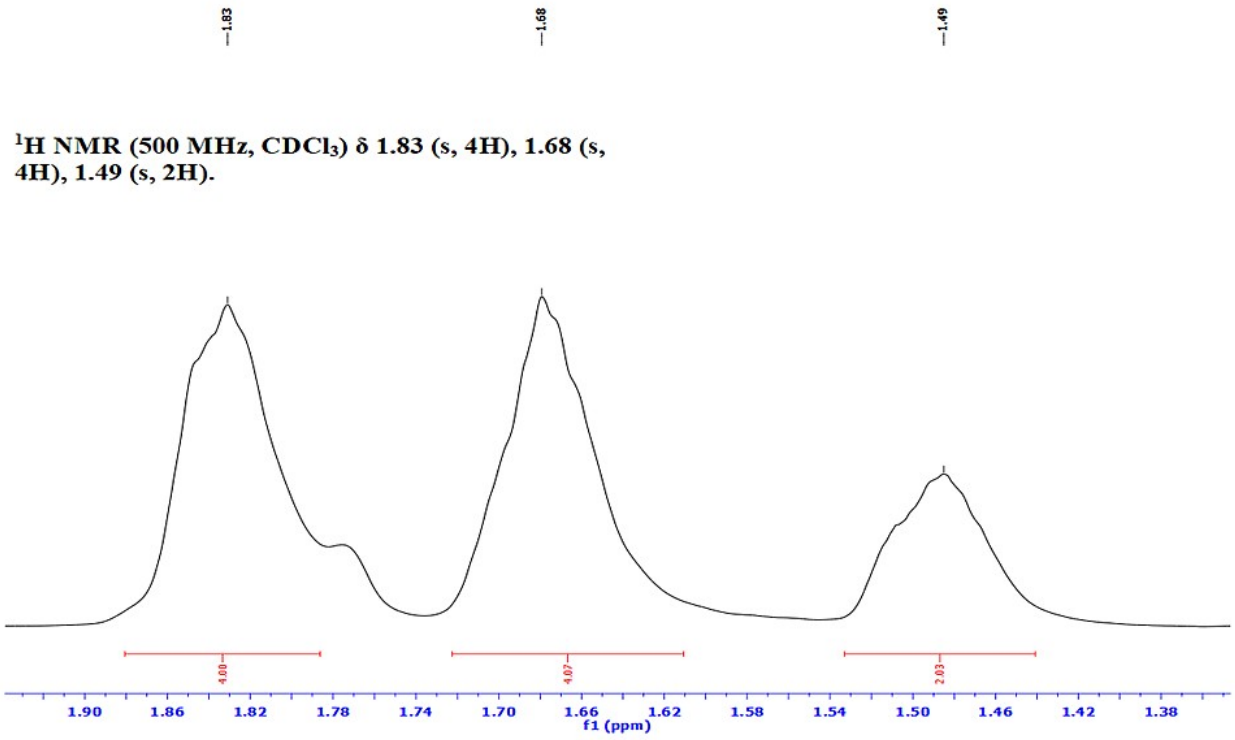

Fig. S13a. ^{1}H NMR Spectrum of the generated cyclohexanol using Pd@SiO_2.

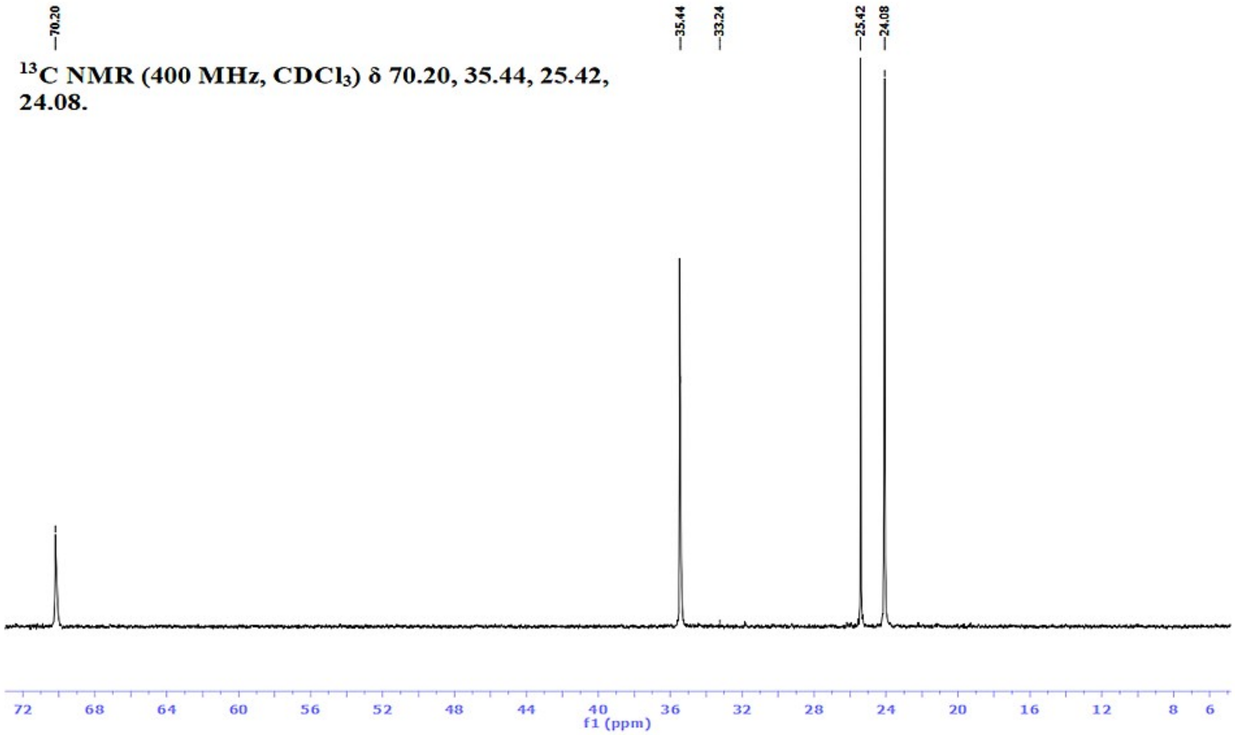

Fig. S13b. ^{13}C NMR Spectrum of the generated cyclohexanol using Pd@SiO_2.

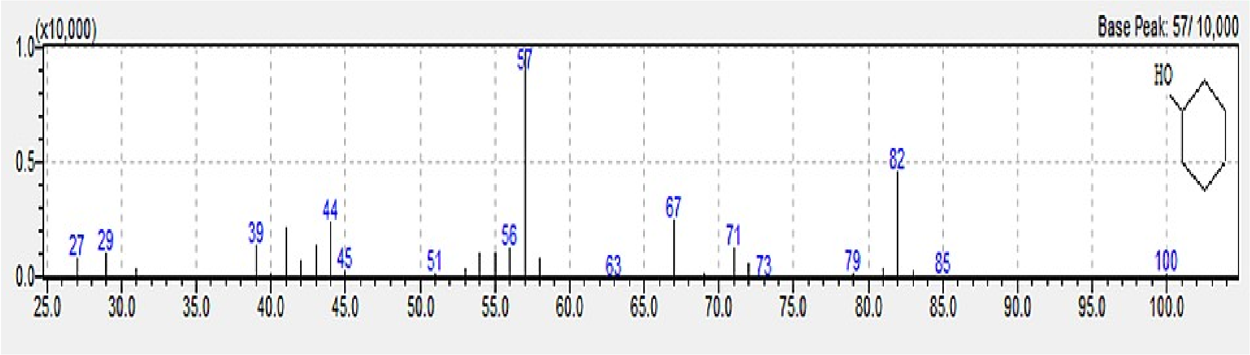

Fig. S14. Product chromatogram of the GC-MS analysis obtained for the hydrogenation of 3-cyclohexanone at 80 °C.

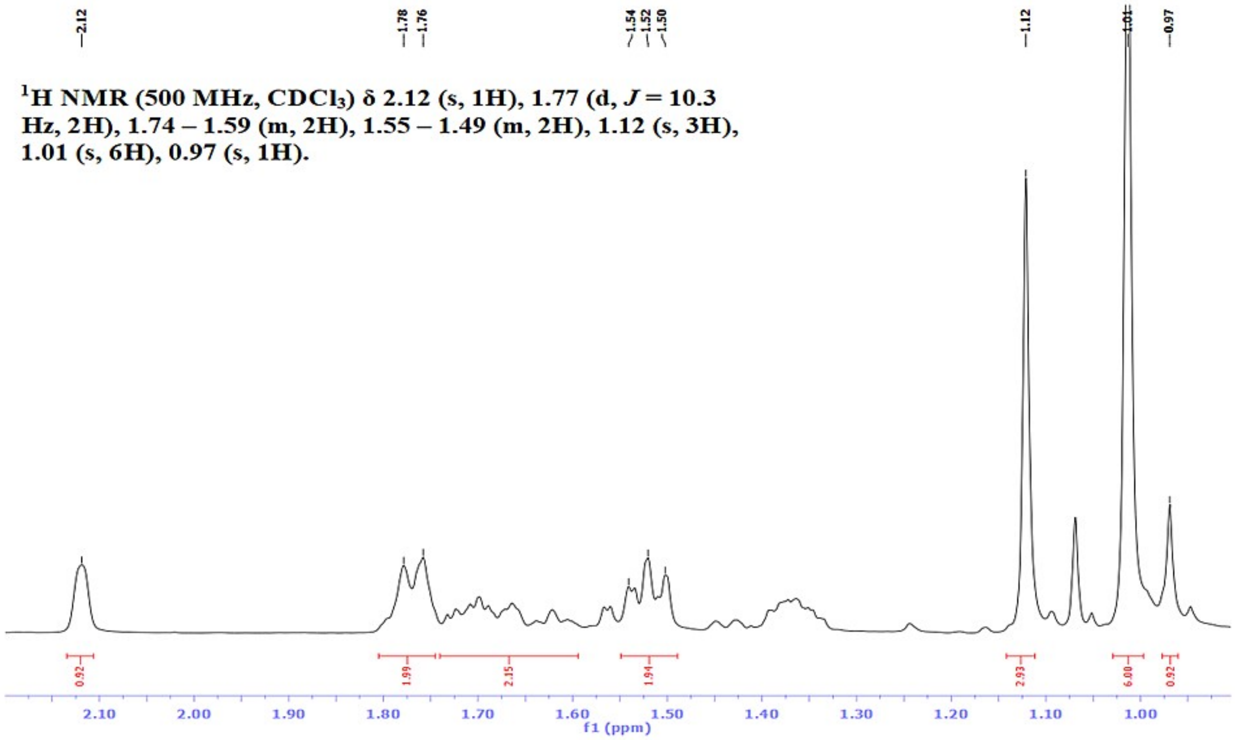

Fig. S15a. ^{1}H NMR Spectrum of the generated fenchol using Pd@SiO_2.

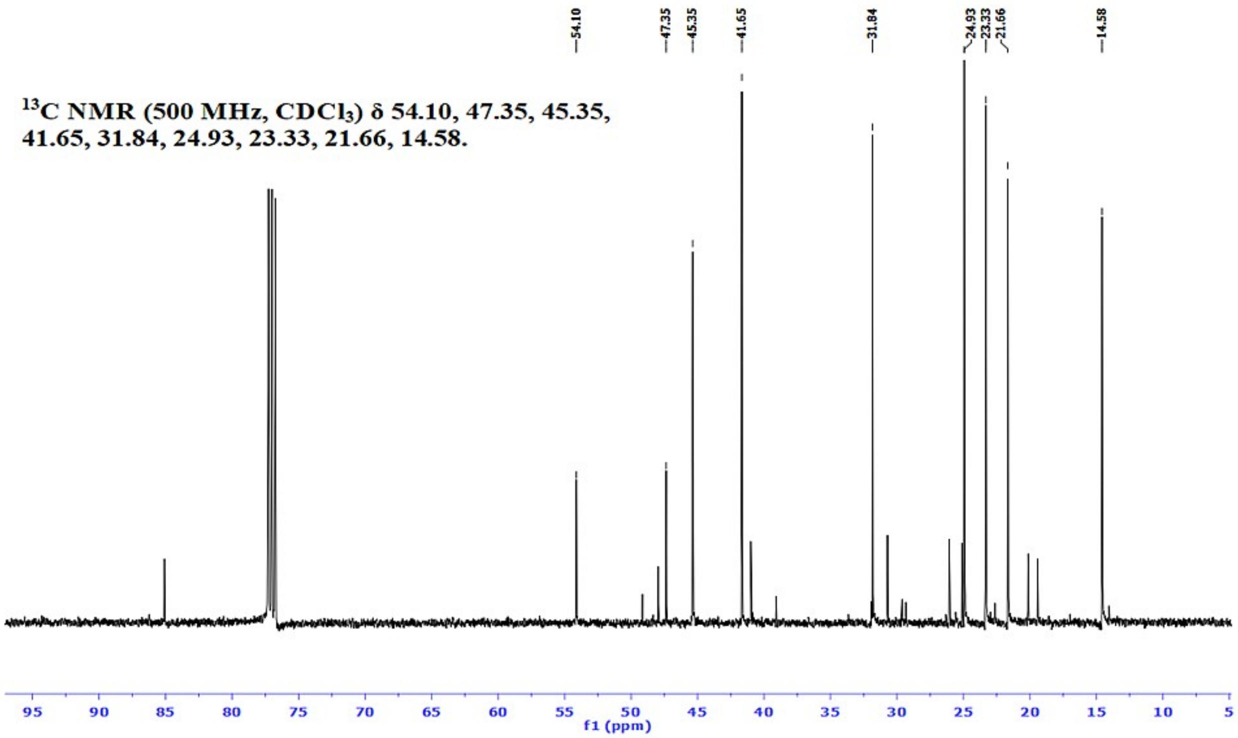

Fig. S15b. ^{13}C NMR Spectrum of the generated fenchol using Pd@SiO_2.

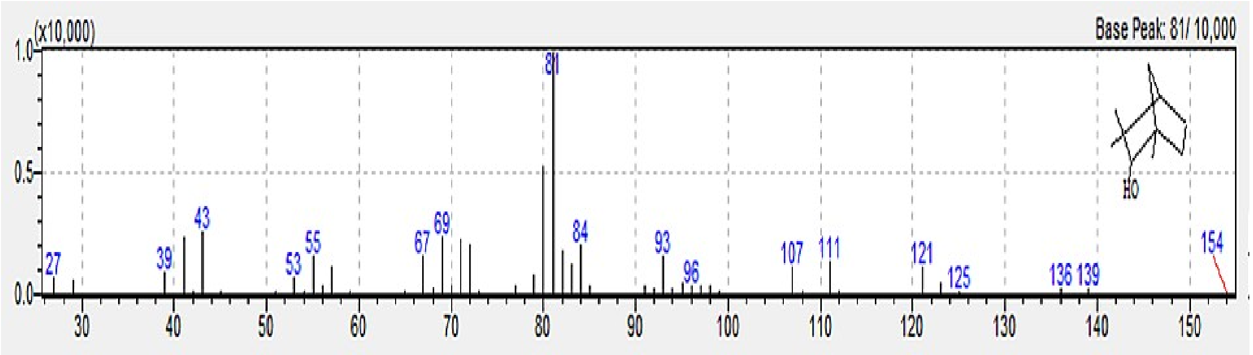

Fig. S16. Product chromatogram of the GC-MS analysis obtained for the hydrogenation of 3-Fenchone at 80 °C.

References

1 A. Balouch, A. Ali Umar, A. A. Shah, M. Mat Salleh and M. Oyama, *ACS Appl. Mater. Interfaces*, 2013, **5**, 9843–9849.

2 M. T. Shah, A. Balouch, K. Rajar, Sirajuddin, I. A. Brohi and A. A. Umar, *ACS Appl. Mater. Interfaces*, 2015, **7**, 6480–6489.

3 D. C. Costa, J. F. Bengoa, S. G. Marchetti and V. Vetere, *Catal. Today*, , DOI:10.1016/j.cattod.2020.09.012.

4 S. R. More and G. D. Yadav, *ACS omega*, 2018, **3**, 7124–7132.

5 J. Y. Kim, W. K. Shin, A. K. Jaladi and D. K. An, *Tetrahedron*, 2018, **74**, 4236–4241.

6 X. B. Zhang, W. N. Lu, J. L. Wang, B. H. Liao, Y. H. Qin, Y. J. Zhang, B. Zhang and S. A. Xin, *Catal. Commun.*, 2019, **119**, 124–128.
